# A Realist Evaluation of the Implementation and Use of Patient‐Reported Outcomes in Four Value‐Based Healthcare Programmes

**DOI:** 10.1111/jan.70018

**Published:** 2025-07-28

**Authors:** Mayara S. Bianchim, Ellie Crane, Leah McLaughlin, Carys Stringer, Gareth Roberts, Adele Cahill, Jane Noyes

**Affiliations:** ^1^ School of Health Sciences Bangor University Bangor UK; ^2^ Aneurin Bevan University Health Board Caerleon UK; ^3^ Welsh Value in Health Centre Swansea UK

**Keywords:** cataract surgery, epilepsy, heart failure, nursing, Parkinson's disease, patient reported outcome measures, realist evaluation, value‐based healthcare

## Abstract

**Aim:**

To investigate what works when using Patient‐Reported Outcome Measures (PROMs), for whom, in what contexts, and why in four Value‐Based Healthcare (VBHC) programmes.

**Design:**

Realist evaluation.

**Methods:**

Evaluation of Heart Failure, Parkinson's Disease, Epilepsy and Cataract surgery programmes using data from a scoping review, documentary analysis, questionnaires, quantitative routinely collected data and semi‐structured interviews with staff, patients and carers (July 2022–August 2023). Programme theories and logic models were developed, tested and refined.

**Results:**

We conducted 105 interviews (67 patients, 21 carers and 17 staff) and collected data from 230 patients (66 Epilepsy, 140 Heart Failure and 24 Parkinson's Disease) and 14 staff via questionnaires. Clinicians used PROMs data to regularly monitor patients with Heart Failure and Epilepsy, which resulted in better triage and tailoring treatment, prioritisation of access based on the urgency of need, and facilitation of referral to relevant professionals. In Heart Failure, this further resulted in a more efficient provision of care and better use of resources, care closer to home, improved health outcomes (e.g., better symptom management) and service redesign. The same was not observed in Epilepsy, as patients who required mental health treatment had to be referred, but they were not always able to access specialist services. PROMs were discontinued in Cataract surgery services mainly due to the lack of integrated IT systems, which caused an increased workload and staff resistance. In Parkinson's Disease, patients were asked to complete PROMs even though the information was not consistently being used.

**Conclusions:**

Findings challenge the orthodoxy that implementing PROMs is universally good and brings about real improvements in patient outcomes in a VBHC context. PROMs are generally ill‐suited for long‐term use with patients in routine care without further adaptation. Greater staff and patient involvement are imperative to enhance the acceptability and relevance of the programmes.

**Implications for the Profession and/or Patient Care:**

Patient‐Reported Outcome Measures can improve care when embedded in well‐supported systems. Implementation must be realistic, involve staff and patients, and be underpinned by clear leadership and robust digital infrastructure. Co‐designed patient‐facing tools can improve accessibility and engagement.

**Impact:**

What problem did the study address? There is limited evidence on how Patient‐Reported Outcome Measures function across different routine healthcare contexts. What were the main findings? Patient‐Reported Outcome Measures improved care in Heart Failure but not in other services, largely due to contextual barriers. Where and on whom will the research have an impact? Findings are relevant for clinicians, service designers, and policymakers seeking to implement meaningful person‐centred outcome measurement in long‐term conditions.

**Reporting Method:**

We adhered to Realist and Meta‐narrative Evidence Syntheses: Evolving Standards II guidance and to the Guidance for Reporting Involvement of Patients and the Public.

**Patient or Public Contribution:**

The study was developed alongside a wide range of patient and public stakeholders involved in the Aneurin Bevan University Health Board Value‐Based Healthcare programme, third sector and specific individuals and groups representing the four included services (i.e., St. David's Hospice Care, British Heart Foundation, Digital Communities Wales, Epilepsy Action, Digital Communities Wales, Parkinson's UK Cymru, Race Equality First, Aneurin Bevan Community Health Council, Value‐ Based Healthcare Patient Reference Group and Wales Council of the Blind). A total of 10 virtual meetings were strategically planned to address gaps, assist in the interpretation of findings, and ensure that outcomes were pertinent and accessible to the specific needs and circumstances of under‐represented or vulnerable groups.

**Summary**

- What is already known?
  ○ Patient‐Reported Outcome Measures are increasingly used in Value‐Based Healthcare to monitor outcomes and inform care decisions.
  ○ Patient‐Reported Outcome Measures are often designed for research contexts and do not easily translate into routine clinical practice without adaptation.
  ○ Implementation of Patient‐Reported Outcome Measures can be hindered by poor IT integration, lack of staff engagement, and limited accessibility for diverse patient groups.
- What this paper adds?
  ○ Patient Reported Outcome Measures contributed to improved triage, prioritisation, and treatment tailoring in Heart Failure services, particularly when led by motivated nursing teams and supported by effective infrastructure.
  ○ Patient‐Reported Outcome Measures were underutilised in some services due to system‐level barriers including IT limitations, workload concerns, and limited clinical integration.
  ○ Patient‐Reported Outcome Measures in Epilepsy highlighted a mismatch between patient needs and service capacity, particularly in accessing appropriate mental health support, limiting their impact on outcomes.
- Implications for practice and/or policy
  ○ Successful Patient‐Reported Outcome Measure implementation requires co‐design with patients, clear leadership, defined responsibilities, and integrated digital systems.
  ○ Patient‐Reported Outcome Measures can enhance care quality when used meaningfully by clinicians and patients; however, they are not universally effective and must be adapted to context.
  ○ Value‐Based Healthcare programmes should be continuously evaluated.
- What does this paper contribute to the wider global clinical community?
  ○ We identified five critical success factors that global clinical decision‐makers and programme leads need to take into account when designing and implementing their programmes.
  ○ Value‐Based Healthcare programmes are expensive and resource‐intensive to implement and may not always deliver the anticipated return on investment when using Patient‐Reported Outcome Measures.
  ○ Value‐Based Healthcare programmes that use Patient‐Reported Outcome Measures and provide limited or no benefit need reform or potential discontinuation.

## 1 Introduction

Value‐Based Healthcare (VBHC) is a model of delivering care focused on improving patient health outcomes within the resources available (Teisberg et al. 2020; Withers et al. 2021). VBHC programmes ensure resources are used in an equitable, sustainable and transparent way to achieve better outcomes for patients (Hurst et al. 2019). These programmes are designed to create value by improving both patient and healthcare professional experiences of care, improving patient health outcomes and reducing the per capita cost of care (Teisberg et al. 2020; Withers et al. 2021). In the face of rising global healthcare demands and costs, VBHC emerges as a promising solution to reconcile the growing need for healthcare with the capacity of health systems to meet the needs of the population (Teisberg et al. 2020; Withers et al. 2021).

VBHC programmes measure value in care by patient outcomes rather than the volume of service delivered (Withers et al. 2021; Eijsink et al. 2023). This model of care uses Patient‐Reported Outcome Measures (PROMs) (Withers et al. 2021; Black 2013) to capture outcomes that matter to patients and improve communication and shared decision‐making, ensuring that patients and healthcare professionals work together whilst considering patients' needs and preferences (Jani and Gray 2015). Using PROMs in routine care also aligns with increasing recognition of the importance of involving patients in their care (Bombard et al. 2018). Implementing PROMs as part of routine care has the potential to provide better monitoring of health status and symptoms, improve personalised care and tailoring of treatment pathways, facilitate shared decision‐making, and ultimately improve health outcomes for patients (Teisberg et al. 2020; Withers et al. 2021; Eijsink et al. 2023). However, there is a dearth of evidence evaluating whether PROMs are effective in maximising value for patients and health services whilst improving outcomes for patients and services (Boyce et al. 2014).

## 2 Background

The implementation and use of PROMs in routine healthcare is commonly nurse‐led (Teisberg et al. 2020; Withers et al. 2021; Silveira Bianchim et al. 2023). It is crucial to understand whether PROMs work from the perspective of patients, family/carers, healthcare professionals and services. A deeper understanding of factors that can create facilitators and barriers to implementation is also warranted to enable the successful use of PROMs in a VBHC context. For instance, PROMs were initially developed for measuring outcomes in research studies and are generally long and complex instruments that might not always be suitable for routine care (Silveira Bianchim et al. 2023). It is also important to note that the implementation and use of PROMs vary across healthcare systems; private providers often use PROMs for commercial purposes, while public systems such as the United Kingdom (UK) National Health Service (NHS) prioritise clinical outcomes and accountability (Withers et al. 2021). It is also important to establish whether the implementation of PROMs increases healthcare professionals' workload (Silveira Bianchim et al. 2023).

In this paper, we report how the realist evaluation helped advance understanding of the challenges of successfully implementing and using PROMs to achieve the ambitions of VBHC. We evaluated four different VBHC programmes, including three long‐term condition predominantly nurse‐led services (Epilepsy, Heart Failure, and Parkinson's Disease) and a medically‐led planned surgical intervention (Cataract surgery) to explore whether PROMs worked as intended to bring about anticipated outcomes. We also conducted Social Return on Investment (SROI) analyses reported elsewhere (Crane et al. 2024).

### 2.1 The Study

#### 2.1.1 Aim

To evaluate the implementation, collection, use, and outcomes of PROMs in the first adopter health board in Wales (Aneurin Bevan University Health Board [ABUHB]). This realist evaluation was designed to address the following question: What works when using PROMs, for whom, in what contexts, and why in a VBHC context?

## 3 Methods

We conducted a realist evaluation (Tilley and Pawson 1997) following a published protocol (Roberts et al. 2023) and reported using RAMESES II guidance (Wong et al. 2016). The realist methodology recognises that interventions (i.e., PROMs) work differently in various contexts (i.e., VBHC) and for different people. This theory‐driven approach seeks to understand ‘what works, for whom, under what circumstances, and how’ (Tilley and Pawson 1997), and is ideal for evaluating complex interventions such as VBHC programmes using PROMs. The strength of the realist approach lies in its ability to go beyond measuring outcomes by identifying the mechanisms that drive change and how these mechanisms are shaped by contextual factors. This allows for a nuanced understanding of implementation success and helps inform how interventions can be adapted across diverse settings. In this study, the use of realist Context‐Mechanism‐Outcome (CMO) chains provided insights into how PROMs influenced patient care, clinician engagement, and service delivery across multiple services.

Patient and Public Involvement and Engagement (PPIE) was guided by the UK Standards for Public Involvement (Partnership UPISD 2019), and we used the Guidance for Reporting Involvement of Patients and the Public Checklist (GRIPP2) (Staniszewska et al. 2017) to report and the Public Involvement in Research Impact Toolkit (PIRIT) (Marie Curie Research Centre and the Wales Cancer Research Centre 2023) to track impact. PPIE contributors were provided with expenses and financial reimbursement (NIHR, HCRW & HRA 2022; NIHR 2023).

We acknowledge that referring to *‘patients’* and not ‘*people living with x condition’* is considered dehumanising. However, the language associated with PROMs refers to *patients* and as we refer to *patients* so often in this study, we have used ‘*patients*’ as a proxy for ‘*people living with x condition’*.

### 3.1 Setting

We evaluated an intervention (PROMs implementation within VBHC programmes) in four services in the ABUHB in Wales. Further details regarding this Health Board and the selection of the VBHC programmes are provided in Supporting Information S1 ‐ Environment Surrounding the Evaluation. Context is also described in each of the four services in Table 1.

**TABLE 1:** Initial programme theories for the implementation and use of Patient Reported Outcome Measures (PROMs) in four Value‐Based Health Care (VBHC) programmes.

VBHC programme	Initial programme theory	Context‐mechanism‐outcomes
Heart Failure	Patient level theory	Patients with access to PROMs (in clinic, over the phone, or online), with sufficient health and digital literacy (for online PROMs), who can access support from clinicians (including reminders and education about the purpose of PROMs and their role in managing symptoms), tailored resources (e.g., translated materials, assistance for older patients with limited mobility or those with cognitive impairments) (context), and who complete PROMs to monitor their symptoms of Heart Failure; the regular PROMs completion can help them develop an increased awareness of their symptoms and overall health status. This heightened awareness can help to validate their concerns and empower them to effectively communicate their needs to healthcare professionals (mechanism), which in turn can help improve patient involvement in positive health‐related behaviours, potentially leading to better self‐management and self‐monitoring, better‐shared decision‐making, and improved health outcomes (e.g., better quality of life, increased life expectancy) (outcomes)
	Clinician level theory	When nurses and other members of the multi‐disciplinary team can access individual PROMs data through systems designed to handle multiple PROMs for the same condition (reducing workflow inefficiencies), have dedicated time to work with these data, receive system‐wide support (including managerial, IT, and financial resources), and are equipped with adequate training on using PROMs and VBHC principles (particularly for tracking symptoms and tailoring care), they are better positioned to engage with and utilise PROMs. Additional factors such as effective communication structures, streamlined referral pathways to specialists, and strong coordination between multidisciplinary teams play a role in optimising PROM implementation and use (context). Nurses and other members of the multi‐disciplinary team utilise PROMs for regular remote monitoring of patient symptoms and needs and this increases their awareness of patients' symptoms through the examination of temporal trends and changes. This increased awareness allows nurses to identify new symptoms or alterations in existing symptoms, resulting in better triage and the ability to tailor treatment and care pathways (i.e., optimisation vs. palliative/complex) based on the severity of the condition (mechanism). This in turn, can help improve nurse‐doctor‐patient communication and potentially lead to a more efficient provision of care, better engagement between doctors, nurses, patients and carers, and optimal resource utilisation. It can also facilitate prioritisation of access based on the urgency of patient needs, reduce waiting time for appropriate treatment, enable seamless referral to relevant professionals and ultimately ensure clinicians are better informed to respond to patients' needs (outcomes)
	Service level theory	When aggregated PROMs data is employed to pinpoint areas within the service that experience higher demand, resource constraints, or fall short of meeting patient expectations—with the support of system‐wide organisational processes such as effective data analysis infrastructure, leadership commitment to data‐driven decision‐making, and communication pathways for feedback and resource planning (context) —services can then identify opportunities for resource allocation/redistribution and quality improvements (mechanism). This in turn, can lead to better management and use of resources, decreased waiting lists, and service redesign with services better able to respond to patient's needs and expectations (outcomes)
Epilepsy	Patient level theory	Patients with access to PROMs (in clinic, over the phone, or online), sufficient health and digital literacy (for online PROMs), support from clinicians (including reminders, education about the purpose of PROMs, and follow‐up feedback on their results), and access to tailored resources (e.g., translated materials, assistance for patients with cognitive impairments or physical disabilities) (context), complete PROMs to monitor their physical and mental health. This increase in awareness of their mental health, can help to validate their concerns and empower them to effectively communicate their needs to nurses and wider healthcare professionals (mechanism), which in turn can help improve patient involvement in positive health‐related behaviours, leading to better self‐management and self‐monitoring, better‐shared decision‐making, and improved patient outcomes (e.g., better Epilepsy control, less anxiety and depression) (outcomes)

	Clinician level theory	When nurses and other members of the multi‐disciplinary team can easily access individual PROMs patient data through well‐functioning digital systems, have dedicated time to work with these data, receive system‐wide support from managerial, IT, and financial resources, and are equipped with adequate training and knowledge of PROMs within VBHC (including its role in shared decision‐making and mental health monitoring), they are better positioned to engage with and utilise PROMs. Additional factors such as effective communication structures within and across services, streamlined referral pathways (especially for mental health services), and a supportive organisational culture also play a role in optimising PROM implementation and use (context). Regular use of PROM data increases their awareness through the examination of temporal trends and changes. This increased awareness can help nurses entify new or worsening mood disorders/seizures and whether the condition is well controlled by medication, potentially resulting in better triage (e.g., signposting to an online cognitive behavioural therapy intervention or a mental health service) and the ability to tailor treatment and medication based on the specific needs of each patient (mechanism). This in turn, can lead to a more efficient provision of care and optimal resource utilisation. It can also facilitate prioritisation of access based on the urgency of patient needs, enables seamless referral to relevant professionals, encourage care closer to home, and ultimately ensure clinicians are better informed to respond to patients' needs (e.g., better informed to adjust medication timing and dosage) (outcomes)
Service level theory	When aggregated PROMs data is employed to pinpoint areas within the service that experience higher demand, resource constraints, or fall short of meeting patient expectations—supported by system‐wide organisational processes such as effective data analysis infrastructure, leadership commitment to data‐driven decision‐making, communication pathways for feedback and resource planning, and the availability of appropriate services to address mental health needs identified by PROMs (e.g., timely referral to mental health services or access to psychological support) (context), this information can serve as a basis for resource redistribution (mechanism). This in turn, can lead to better management and use of resources, decreased waiting lists, and service redesign with services better able to respond to patient's needs and expectations (outcomes)
Parkinson's Disease	Patient level theory	Patients with access to PROMs (in clinic, over the phone, or online), sufficient health and digital literacy (for online PROMs), support from clinicians (including reminders and education about PROMs), and access to tailored resources that accommodate complex needs (e.g., assistance for patients with motor impairments, severe physical symptoms, or cognitive decline) (context), complete PROMs to monitor their Parkinson's Disease symptoms. Regular PROMs completion can help them develop an increased awareness of their symptoms and overall health status. This heightened awareness can serve to validate their concerns and empower them to effectively communicate their needs to nurses and wider healthcare professionals (mechanism), which in turn can help improve patient involvement in positive health‐related behaviours, potentially leading to better self‐management and self‐monitoring, better‐shared decision‐making, and improved health outcomes (e.g., better quality of life, increase or maintained autonomy) (outcomes)

	Clinician level theory	When nurses and other members of the multi‐disciplinary team have access to individual PROMs data that is timely and aligned with patient appointments (avoiding delays that lead to outdated data), have dedicated time to review and act on these data, and receive system‐wide support (including managerial, IT, and financial resources), they are better positioned to engage with and utilise PROMs. Adequate training on VBHC principles and PROMs for complex cases (e.g., patients with cognitive or motor impairments), effective communication between services, and referral pathways for specialised care also play crucial roles in optimising PROM implementation and use (context). Nurses and other members of the multi‐disciplinary team utilise PROMs for regular remote monitoring of patient symptoms and needs and this increases their awareness of patients' symptoms through the examination of temporal trends and changes. This increased awareness can help nurses to identify new symptoms or alterations in existing symptoms, resulting in better triage and the ability to tailor treatment and care based on the specific needs of each patient (mechanism). This in turn can lead to a more efficient provision of care and optimal resource utilisation. It can help facilitate prioritisation of access based on the urgency of patient needs, enables seamless referral to relevant professionals, encourages care closer to home, and ultimately ensures clinicians are better informed to respond to patients' needs (e.g., better informed to manage new or worsening symptoms and changes in the condition long term) (outcomes)
Service level theory	When aggregated PROMs data is employed to pinpoint areas within the service that experience higher demand, resource constraints, or fall short of meeting patient expectations— with the support of system‐wide organisational processes such as effective data analysis infrastructure, leadership commitment to data‐driven decision‐making, and communication pathways for feedback and resource planning (context)— this information can serve as a basis for resource redistribution (mechanism). This in turn, can lead to better management and use of resources, decreased waiting lists, and service redesign with services better able to respond to patient's needs and expectations (outcomes)
Cataract surgery	Patient level theory	Patients with access to PROMs (often completed on paper due to online system limitations) require sufficient health literacy to understand and respond to the questions, support from clinicians (including reminders and education about PROMs and their role in surgical decision‐making), and access to tailored resources. Many patients may experience visual impairments, including partial or total blindness, and may require assistance from family members, carers, or healthcare staff to complete PROMs (context). Patients who complete PROMs to triage for Cataract surgery can develop an increased awareness of their Cataract symptoms. This heightened awareness can serve to validate their concerns and empowers them to effectively communicate their needs to healthcare providers (mechanism), which in turn can help patients to be more involved and informed in deciding whether to have surgery or not leading to improved outcomes for patients (e.g., better visual related quality of life) (outcomes)
	Clinician level theory	When eye surgeons can access PROMs data that is readily and timely available—with the support of system‐wide infrastructure such as administrative staff, IT resources, integrated digital systems, effective communication structures, and streamlined processes for managing surgical waiting lists and referrals (context)—they can examine relevant patient information (e.g., severity of symptoms, patient age, living situation, and comorbidities). This can help facilitate evidence‐informed and timely triage based on the urgency of patient needs (mechanism), resulting in improved resource utilisation, reduced waiting lists, and clinicians being better informed to prioritise and plan cataract surgeries according to clinical needs (outcomes)

Service level theory	When system‐wide processes and support (including administrative staff, IT infrastructure, and integration of PROMs data into clinical systems) ensure that aggregated PROMs data is timely, reliable, and accessible, services can use this data to identify areas of high demand and unmet patient needs, enabling triage for cataract surgery based on patients' needs and Disease severity (context). This can support more efficient allocation of resources, with prioritisation of patients according to clinical urgency and individual circumstances (mechanism), which in turn can lead to improved resource management, reduced waiting lists, and service redesign that enhances the service's ability to meet patients' needs and expectations (outcomes)

### 3.2 Data Collection and Analysis

Data were collected between July 2022 and August 2023. Data collection and analyses included diverse data sources (Table 2 and [Link], [Link], [Link]).

**TABLE 2:** Data collection and analysis methods.

Data source	Method of analysis
*Documentary analysis* of 22 VBHC documents, including audits, reports, implementation documents and policy documents, aimed at identifying essential elements for developing initial programme theories	We developed a coding framework tailored to explore the programme theories and their context as they were initially intended to work at the beginning of the implementation of the programmes. Data relevant to context, mechanisms and outcomes of the use of PROMs, in general and specifically in each programme were coded on NVivo 12 (QSR International Ltd., Australia) and analysed using content analysis. A report was produced summarising how each programme was designed to work along with process maps (Supporting Information S2 ‐ Process Maps) outlining the use of PROMs in each service
*Realist exploratory semi‐structured interviews*. VBHC network staff, healthcare professionals from the services, patients, and their carers. Interviews, lasting up to 60 min, were conducted either over the phone or in person by three researchers (LM, MSB, BN). Specific interview schedules were prepared for patients and staff of each service (Supporting Information S3 ‐ Example Topic Guide) Patients and carers were purposefully selected based on age, gender, ethnicity, service, and PROM completion status. Initially, ABUHB staff approached 411 patients (142 Epilepsy, 87 Heart Failure, 49 Parkinson's Disease, and 133 Cataract surgery) to inquire whether they would consent to be contacted for interviews. Recruitment methods included sending invitations through the Health Board's internal PROMs platform, scheduling routine appointments, contacting via email, or sending invitations by post. Patients returned consent to contact forms in prepaid envelopes, after which the research team contacted patients to schedule the interviews. Carers or family members were recruited through these patients. Staff assisted potential participants when necessary, and consultees were assigned to individuals lacking the capacity to consent. Twenty‐seven potential participants declined to participate and 14 were unreachable (Supporting Information S4 ‐ Reasons for not Participating in Interview)	Analysis of interviews was an ongoing iterative process within a wider configurational explanation (CMO). Interviews were recorded, transcribed, and imported into NVivo 12 (QSR International Ltd., Australia) for analysis. A coding framework based on CMO configurations was used. Data were then analysed using realist configurational analysis, identifying contexts, mechanisms, outcomes and their interactions to build, test and/or refine programme theory
*Online surveys*. Six online surveys were developed and distributed to both patients and healthcare professionals across the three nurse‐led services. Patients were included in the survey if (1) they were verified patients in the Heart Failure, Epilepsy or Parkinson's Disease services, and (2) had completed PROMs in the past 2 years as part of their care. Staff were included in the survey if (1) they worked in Heart Failure, Epilepsy or Parkinson's Disease services and (2) had experienced using PROMs. A total of 3577 patients (2388 Heart Failure, 884 Epilepsy, and 305 Parkinson's Disease) received invitations to complete online questionnaires via text message, with a follow‐up reminder sent a week later. Patients were offered a £5 voucher for their participation. Questionnaires covered four domains: (1) demographic information, (2) achievement of outcomes, (3) the role of PROMs in achieving each outcome, and (4) outcomes that were meaningful for patients. Fourteen staff members, including nurses, specialist nurses, medical consultants, and nursing assistants (6 Heart Failure, 5 Epilepsy and 3 Parkinson's Disease), were invited via email or phone	Statistical analysis was conducted using SPSS software (SPSS Inc., Chicago, Ill., USA) to calculate frequencies and perform descriptive analysis of the online survey data
*Routinely collected service data* including average waiting times for initial appointments and follow‐ups, average waiting times for referrals, number of patients referred to specialist mental health services and PROMs completion rates	Routine data were analysed using SPSS software (SPSS Inc., Chicago, IL, USA) to generate descriptive statistics where applicable
*Scoping review* (Silveira Bianchim et al. 2023)	Forty‐three studies were included using the five‐stage framework approach alongside the scoping review method described by Arksey and O'Malley (Arksey and O'Malley 2005)
*Fieldnotes and reflections*	Were recorded, transcribed, and imported into NVivo 12 (QSR International Ltd., Australia) and analysed along with data from the realist interviews
*Patient and public involvement and engagement (PPIE)*. The study was developed alongside a wide range of PPIE stakeholders. See PPIE statement for a detailed description.	Tailored sessions were conducted for each group, considering their backgrounds, and lived experiences. Ten virtual meetings were held to address gaps, interpret findings, and ensure outcomes were relevant to under‐represented or vulnerable groups. PPIE contributors were involved in tasks such as the scoping review, developing patient online surveys, formulating initial programme theories and logic models, and adapting CMO chains to meet the needs of vulnerable populations in Wales.

Abbreviations: ABUHB, Aneurin Bevan University Health Board; CMO, Context‐Mechanism‐Outcome; PROMs, Patient‐Reported Outcome Measures; VBHC, Value‐Based Health Care.

Inclusion criteria for interviews and questionnaires

- Stakeholders involved in PROMs and VBHC programmes in ABUHB and other relevant health boards in Wales.
- All ABUHB professionals involved in routine PROMs collection in selected VBHC programmes.
- Patients aged over 18 in selected VBHC programmes
- Carers over 18 linked to patients receiving care in selected VBHC programmes
- Consultees for individuals lacking mental capacity to consent.

Exclusion criteria

- Individuals under 18.
- Individuals without mental capacity for whom a consultee was not available

### 3.3 Realist Analysis

#### 3.3.1 Developing Initial Programme Theories

Initial programme theories and logic models (Supporting Information S5 ‐ Logic Models) explaining how the integration of PROMs in routine care in VBHC programmes was formulated drawing on the documentary analysis, a scoping review (Silveira Bianchim et al. 2023), and early staff and PPIE. Additionally, process maps were created to illustrate the logic and process of implementation before, during, and after the COVID‐19 pandemic (Supporting Information S2 ‐ Process Maps).

##### 3.3.1.1 Testing and Further Refining the Initial Programme Theories

Five researchers [JN, GR, LM, MSB, EC] analysed data relevant to all four programmes by using the recommended structure to organise and interpret evidence: context (IF), mechanism (THEN), outcome (LEADING TO). Additional clarification was sought from AC and other members of the central and local VBHC teams. Two authors [MSB, JN] then mapped the initial programme theories against all data sources to create CMO chains. This process consisted of testing each outcome in the programme theory and identifying the type of failure (i.e., partial or complete theory failure, implementation failure, adherence & engagement failure) and where in the process a failure happened (early or later in the causal pathway, or different causal pathway). Consistent with realist approaches, analysis was retroductive in that it oscillated between inductive and deductive logic to multiple data sources, with key PPIE and stakeholders involved during this process.

We also reviewed the realist findings for the VBHC programmes (including programme theories and CMO configurations) ‘vertically’ to identify common thematic elements according to CMOs. Data were also analysed across each service ‘horizontally’ to uncover potential generative causal patterns between mechanisms and outcomes. We mapped the actual process of implementation (work as done) compared with how it was planned (work as imagined). We also translated CMOs into generalisable logic models (middle‐range theories) for implementing VBHC programmes using PROMs at scale. This process translated the specifics of implementing VBHC and PROMs in the selected services to more analytically driven generalisable theories for scaling up the benefits from these care delivery models to achieve large system transformation across health boards in Wales and beyond.

### 3.4 Ethical Considerations

The study was approved by the Wales Research Ethics Committee #5 on March 2022 (22.WA/0044). Informed consent was received from all participants prior to the interviews and online questionnaires. Further details of the ethical safeguards put in place can be found in the published protocol (Roberts et al. 2023).

#### 3.4.1 Rigour and Reflexivity

The realist evaluation was rigorously conducted according to published realist standards using a wide range of data sources and extensive PPIE based on the UK standards (Chief Scientist Office HaCRW 2019). The multi‐disciplinary research team included experts in VBHC, nurses, doctors, a physiotherapist, health services researchers and patient and public representatives. We met regularly, virtually and face to face, to discuss emerging findings and reflect on potential biases that could impact interpretation. Although we worked very closely with the VBHC team, all researchers were independent and employed by an external organisation.

## 4 Results

**FIGURE 1:**
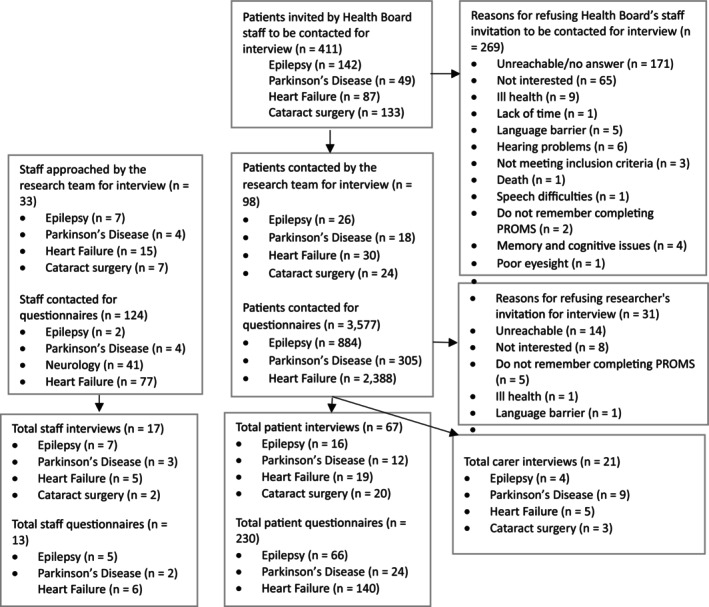
Patients, carers and staff recruitment for interviews and questionnaires flowchart.

We conducted 105 realist interviews (28 Epilepsy, 26 Heart Failure, 27 Parkinson's Disease and 24 Cataract surgery). These interviews involved 67 patients, 21 family member carers, and 17 staff, including medical consultants, specialist nurses, nurses and nursing assistants (Figure 1). Among the patients, 29 (43%) were female, and the majority of participants were married and lived with their spouse (*n* = 30) (Table 3). Completion rates of PROMs varied between conditions: only about half of the patients with Epilepsy (51%) and Parkinson's Disease (43%) were completing them at the time of the interviews, whereas around three‐quarters of patients with Heart Failure were completing PROMs (approximately 73% in 2023).

**TABLE 3:** Demographics of patients and carers interviewed for the evaluation of two Value‐Based Healthcare Programmes.

Patient demographics (*n*)	Heart failure (*n* = 19)	Epilepsy (*n* = 16)	Parkinson's Disease (*n* = 12)	Cataract surgery (*n* = 20)
Sex *n* (%)				
Female	8 (42%)	9 (56%)	3 (25%)	9 (45%)
Male	11 (58%)	7 (44%)	9 (75%)	11 (55%)
Age *n* (%)				
18–35	0 (0%)	5 (31%)	0 (0%)	0 (0%)
36–50	1 (5%)	4 (25%)	1 (8%)	1 (5%)
51–70	6 (32%)	6 (38%)	4 (34%)	8 (40%)
> 71	12 (63%)	1 (6%)	7 (58%)	11 (55%)
Relationship status *n* (%)				
Single	5 (27%)	3 (19%)	4 (34%)	5 (25%)
Co‐habiting	1 (5%)	2 (12.5%)	2 (16%)	2 (10%)
Married or civil partnership	10 (53%)	8 (50%)	4 (34%)	8 (40%)
Divorced	2 (10%)	2 (12.5%)	2 (16.7%)	2 (10%)
Widowed	1 (5%)	1 (6%)	0 (0%)	3 (15%)
Ethnicity *n* (%)				
White	17 (90%)	12 (75%)	11 (92%)	14 (70%)
Black/African/Caribbean	0 (0%)	1 (6%)	0 (0%)	2 (10%)
Hispanic	0 (0%)	0 (0%)	0 (0%)	0 (0%)
Asian	0 (0%)	0 (0%)	0 (0%)	1 (5%)
Mixed/multiple groups	0 (0%)	0 (0%)	1 (8%)	0 (0%)
Unknown	2 (10%)	3 (19%)	0 (0%)	3 (15%)
Employment *n* (%)				
Working	1 (5%)	7 (44%)	2 (16.7%)	5 (25%)
Unemployed	1 (5%)	4 (25%)	1 (8%)	1 (5%)
Retired	17 (90%)	5 (31%)	9 (75.3%)	14 (70%)

Carers demographics (*n*)	Heart failure (*n* = 5)	Epilepsy (*n* = 4)	Parkinson's disease (*n* = 9)	Cataract surgery (*n* = 3)
Sex *n* (%)				
Female	4 (80%)	3 (75%)	5 (55.5%)	2 (66.7%)
Male	1 (20%)	1 (25%)	4 (44.5%)	1 (33.3%)
Age *n* (%)				
18–35	0 (0%)	0 (0%)	0 (0%)	0 (0%)
36–50		1 (25%)	1 (11.1%)	1 (33.3%)
51–70	2 (40%)	2 (50%)	6 (66.7%)	1 (33.3%)
> 71	1 (20%)	1 (25%)	2 (22.2%)	1 (33.3%)
Relationship status *n* (%)				
Single	0 (0%)	1 (25%)	0 (0%)	1 (33.3%)
Living with a partner	0 (0%)	0 (0%)	0 (0%)	0 (0%)
Married or in civil partnership	1 (20%)	2 (50%)	0 (0%)	2 (66.7%)
Divorced	0 (0%)	1 (25%)	0 (0%)	0 (0%)
Ethnicity *n* (%)				
White	1 (20%)	3 (75%)	0 (0%)	3 (100%)
Black/African/Caribbean	0 (0%)	0 (0%)	0 (0%)	0 (0%)
Hispanic	0 (0%)	0 (0%)	0 (0%)	0 (0%)
Asian	0 (0%)	1 (25%)	0 (0%)	0 (0%)
Mixed/multiple groups	0 (0%)	0 (0%)	0 (0%)	0 (0%)
Relationship to patient *n* (%)				
Wife	2 (40%)	2 (50%)	4 (44.5%)	1 (33.3%)
Husband	0 (0%)	1 (25%)	2 (22.2%)	1 (33.3%)
Sibling	2 (40%)	1 (25%)	0 (0%)	0 (0%)
Sibling‐in‐law	1 (20%)	0 (0%)	0 (0%)	0 (0%)
Son/daughter	0 (0%)	0 (0%)	3 (33.3%)	1 (33.3%)

Abbreviation: PROMs, Patient‐Reported Outcome Measures.

A total of 230 patients (66 Epilepsy, 140 Heart Failure, and 24 Parkinson's Disease) were sent a digital link on their mobile phone to complete an online survey. The patient response rates were 7.9% (*n* = 189) for Heart Failure, 7.5% (*n* = 66) for Epilepsy and 7.9% (*n* = 24) for Parkinson's Disease. Less than half of the patients who completed the online questionnaire recalled completing PROMs, with 23% (*n* = 32) in Heart Failure, 47% (*n* = 31) in Epilepsy and 42% (*n* = 10) in Parkinson's Disease recalling they had completed a recent PROM. Additionally, 14 staff members, including specialist nurses, medical consultants, nurses and nursing assistants, completed an online questionnaire (Supporting Information S6 ‐ Participants Demographics ‐ Questionnaires).

### 4.1 Initial Programme Theories

Initial programme theories are described in Table 1 and annotated logic models for each VBHC programme can be found in Supporting Information S7 ‐ Annotated Logic Models. An example of the IF, THEN, LEADING TO framework used to integrate the different sources of data can be found in Supporting Information S8 ‐ IF, THEN, LEADING TO Framework.

### 4.2 Findings

The Heart Failure service successfully implemented PROMs at the clinician (nurse) and service levels but the programme did not result in patients and nurses working any better together to consider patients' needs and preferences—a core principle of VBHC. Despite successfully utilising PROMs to triage and monitor mood disorders over time, Epilepsy nurses lacked the necessary skills and resources within their service to address low mood in patients identified by PROMs; patients did not consistently complete or value the online mental health resource that they were referred to by automated email. Referral to mental health services for those with very low mood took too long or did not happen as patients were required to request referral via their GP, and mental health services did not have sufficient capacity to accept them. The VBHC programme stopped in Cataract surgery during the evaluation due to IT issues (lack of a functional integrated system). Patient‐level outcomes, including shared decision‐making, self‐monitoring, and self‐management, were not achieved across any of the programmes. We identified overarching contextual and implementation factors that created barriers and facilitators (Table 4) for the use of PROMs as part of VBHC programmes.

**TABLE 4:** Contextual and implementation factors that created barriers and facilitators for the use of Patient‐Reported Outcome Measures (PROMs) as part of a Value‐Based Healthcare (VBHC) strategy in all four tracer conditions.

Factors	Barriers	Facilitators
Digital and technology factors	– Lack of electronic PROM systems that are integrated with patient medical records led to staff resistance and increased workload* – Lack of accessible and well‐functioning digital systems that require limited effort from clinical staff. Digital systems affected: – *Data collection* — Paper‐based PROMs had to be manually entered into an online system separate from the one currently used by the service^Ω^. Staff described that it was not always easy to identify in the system which questionnaire a patient was due when multiple different PROMs questionnaires were used for the same condition^α^ – *Data analysis and reporting* **—** It was not straightforward to input PROMs data captured via phone or paper into the system which hindered PROMs analysis and reporting as staff had to deal with different electronic and paper copies* – These issues caused increased workload and lack of adherence from staff* – Lack of a dashboard at patient level is a possible reason for lack of patient feedback and failure to improve patient self‐management and self‐monitoring^&^ – Lack of reliable internet or equipment (phones or laptops) to access electronic PROMs. Some older patients did not have equipment or an email account to access the electronic questionnaires. PROMs were done via paper or over the phone during appointments in these cases. Staff reported that this was quite time‐consuming and potentially affected PROMs completion*	– Access to a reliable internet positively affected how patients accessed PROMs and PROM completion ratings [some clinics offered to help patients complete over the phone and or paper version]^&^, ^α^, ^β^ – Access to equipment and email account to access and complete electronic PROMs* – Digital literacy*
Factors associated with patients and carers	– Low completion rates. Staff described patients often thought PROMs were spam or they did not understand what or who they were for, which resulted in low adherence from patients^&^ – Some patients, especially older individuals living alone, struggled to access the online platform to complete PROMs* – Patients completing PROMs but did not receive any feedback regarding their scores — we found evidence that patients disengaged PROMs if they did not receive any type of feedback^&^, ^α^ – Lack of communication and patient education on PROMs and VBHC. Patients were not aware that PROMS were part of their care and patients' accounts described they were never told why they were asked to complete PROMs. This resulted in disengagement from patients and low completion rates* – Lack of planning and provisions for patients whose first language was not English, particularly in multicultural locations, which increased inequality and difficulty for minorities to access the intervention* – PROMs not covering outcomes that were important for patients and not following changes in patient's needs over time (e.g., menopause). PROMs were not appropriate for patients with complex needs and multiple comorbidities which resulted in a lack of adherence and low completion rates^&^, ^α^, ^β^ – Patient did not understand the content of the PROMs questions or feedback and were upset over being confronted by their condition, which resulted in a lack of adherence and low completion rates^&^ – Digital literacy, particularly for patients with cognitive impairments, which resulted in a lack of adherence and low completion rates* – PROMs were also unsuitable for patients with complex needs or advanced illnesses, as certain questions were inappropriate given the severity of their symptoms (e.g., blindness, palliative care)*	– Education on VBHC and PROMs helped patients and close family members understand how and why PROMs were being used as part of their routine care* – Reminding patients to complete PROMs increased completion rates and adherence to intervention* – Allowing carers to help patients complete PROMs improved completion rates and adherence to intervention* – Planning for hybrid delivery (digital/paper PROM) or helping patients to complete PROMS during appointments to allow for patient preference and requirements, and to improve retention*
Factors associated with healthcare staff and stakeholders	– Staff resistance resulting from lack of training on using PROMs as part of a VBHC context, lack of IT integration with increased workload for staff* – Management of staff capacity and responsibility in relation to the additional clinical burden of PROMs resulted in a lack of adherence from staff and patients* – Lack of staff understanding of the role of PROMs within a VBHC strategy resulted in failure to achieve improved shared decision‐making and person‐centred care* – Increased staff workload resulted in disengagement of staff with PROMs*	– Staff motivation, engagement, and ownership in the delivery of PROMs helped with adherence* – Provision of dedicated PROMs support staff helped reduce workload for other members of staff and manage administration of PROMs and patient feedback^&^, ^α^ – Staff training and support for clinicians and staff on the following: Staff training on VBHC principles and the role of PROMs within this context. This was essential in ensuring PROMs were implemented as intended and that staff understood the purpose of PROMs, helping to consolidate engagement. It also provided space for staff to voice concerns and co‐produce solutions Staff training on how to use the electronic systems and development of Standard Operating Procedures with the development of a clear process pathway
Structural and organisational factors	– Lack of communication within and between services resulted in failure to accomplish key outcomes (such as improved mental health) following referral of patients^&^ – Lack of resources within a department to address issues raised with PROMs^β^, ^&^. For instance, PROMs were implemented in the Epilepsy service to help track and monitor mood disorders, but an ineffective system was in place within the department to help address these issues, resulted in failure to accomplish key outcomes^&^	– Aligning PROMs timeline to different care pathways, adjusting frequency of data collection when needed^α^ – Dedicated time and resources to implement and deliver PROMs to avoid increased staff workload* – System‐wide institutional support (managerial, IT, financial) to improve adherence and engagement with PROMs* – Ongoing evaluation and iterative refinement of PROMs systems with small incremental changes to allow modifications according to patients' needs to achieve person centred approach* – The implementation of an electronic referral system along with PROMs significantly reduced staff workload^α^

* Across all 4 conditions.

^&^ Epilepsy.

^α^ Heart failure.

^β^ Parkinson's Disease.

^Ω^ Cataract surgery.

**FIGURE 2:**
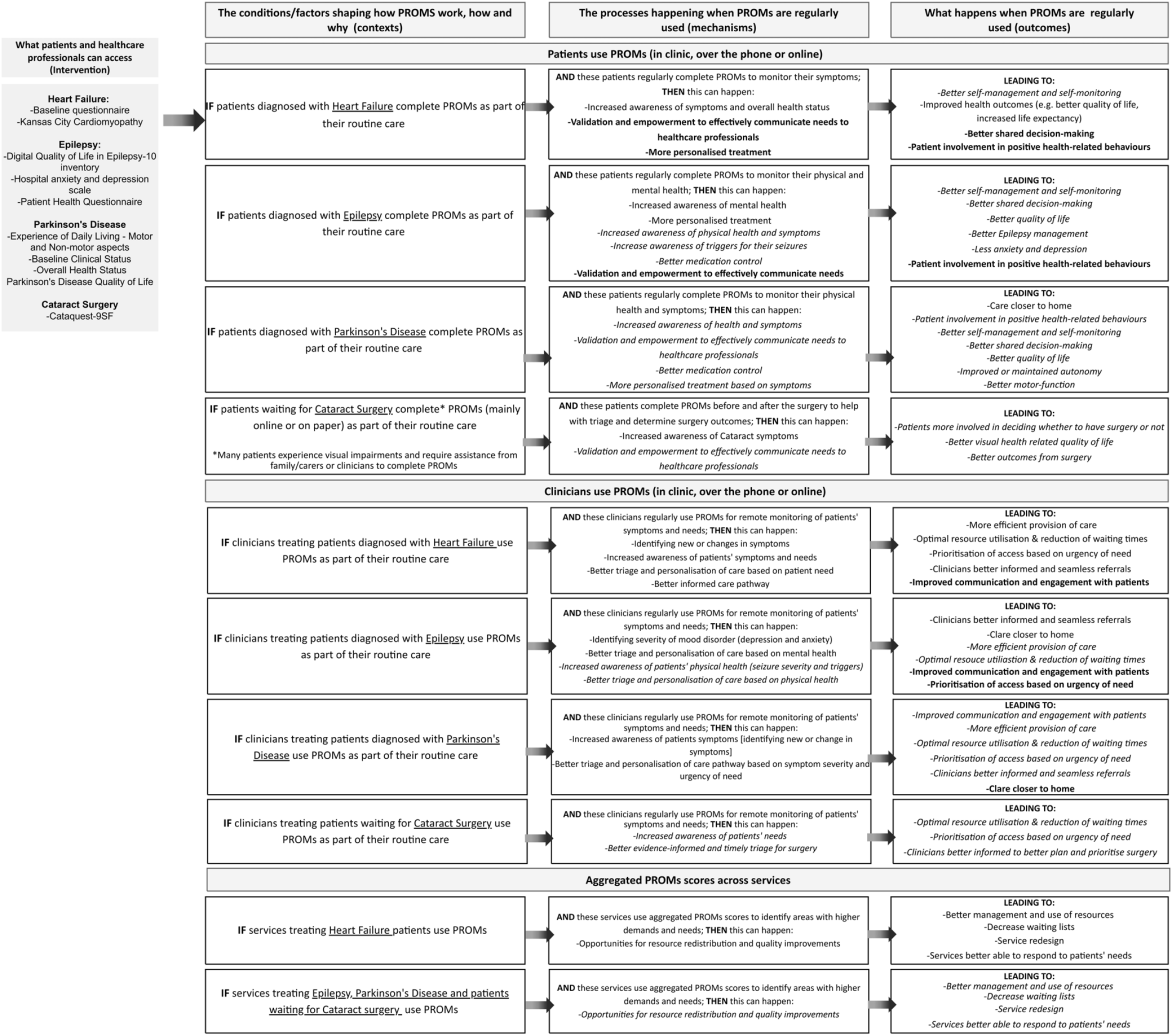
Logic Model illustrating Programme Theories for the use of Patient‐Reported Outcome Measures (PROMs) across four Value‐Based Healthcare Programmes (Heart failure, Epilepsy, Parkinson's Disease and Cataract surgery). Black text shows initial programme theories (IPTs) that were tested and refined based on evidence. Italic text indicates a lack of evidence to test and refine the IPT. Bold text indicates IPTs that could not be tested due to gaps in evidence, but for which insights from patient and public involvement were informative.

In the next section, we outline the programme theories at three levels: (1) patient, (2) clinician and (3) service, as detailed in Table 1 and Figure 2. Following this, we describe the identified CMO configurations.

#### 4.2.1 VBHC Intervention at Clinician Level (CMO Chain 1)

Please refer back to Table 1 for the initial programme theories that were tested. We found that PROMs only worked at the clinical level in Heart Failure and partially worked in Epilepsy (Figure 2).

##### 4.2.1.1 PROMs Used to Monitor Patient's Health, Inform Care Pathways, Redistribution of Resources and Service Redesign

**FIGURE 3:**
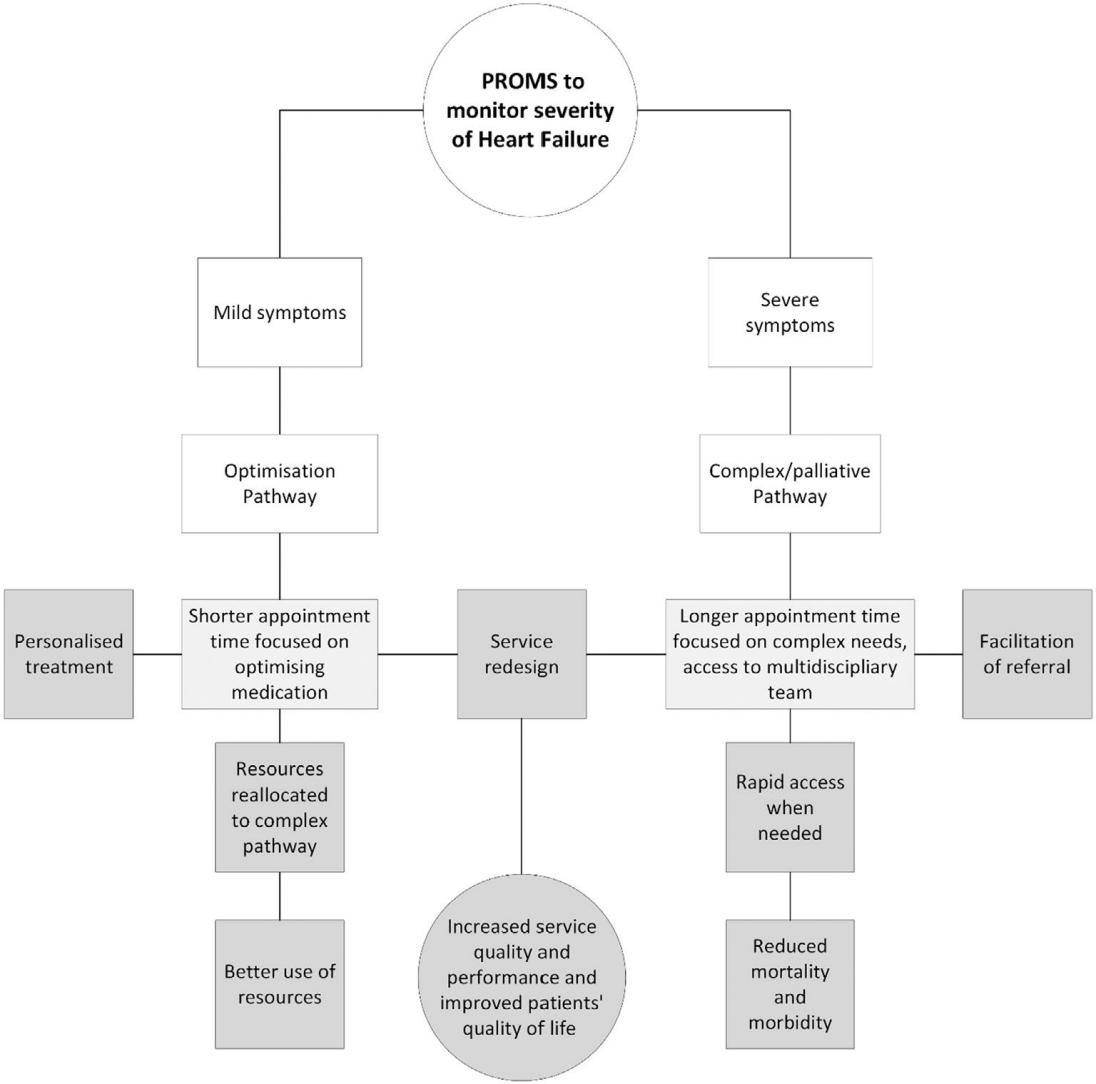
Patient‐Reported Outcome Measures (PROMs) used to inform care pathways.

Heart Failure nursing staff used PROMs to understand the severity of symptoms and decide whether patients should follow an optimisation clinic or enter the complex/palliative care pathway (Figure 3).

Four out of six nurses agreed that the use of PROMs in Heart Failure helped tailor care pathways to patient severity, with everyone (patients and nurses) expressing overall improvement in care. The use of PROMs to inform care pathway selection not only facilitated personalised treatment but also enabled the redistribution of resources to those with more urgent and complex needs. Complex patients with greater severity gained access to a multidisciplinary team, or were referred to palliative care if necessary, resulting in rapid access to treatment and better use of resources. Once the pathway of care was established, some patients in the palliative care pathway felt that PROMs were no longer helpful or appropriate for monitoring their health. For such patients, advanced care planning and a palliative care PROM would likely be more beneficial. Some nurses and PPIEs acknowledged that there was ‘*a lot of work still to be done*’ in the palliative care pathways, particularly regarding the use of PROMs. It is noteworthy that PROMs were not the only instrument used to inform the care pathway in Heart Failure. Nurses also used a traffic light system tool to help inform patients about their symptoms and another clinical tool to identify patients in the palliative care pathway at risk of deterioration.

Integral to the programme was the incorporation of Heart Failure Clinician‐Reported Outcome Measures (CROMs), employed alongside other instruments such as clinical tests, forms, and assessments. CROMs were designed to provide clinical context to symptoms and needs assessed by PROMs, as elucidated below:

With the clinical CROM as well, we can say okay, how much of that symptom burden is related to Heart Failure, and then okay, some of it may be because they've [patients] got, I don't know, hip pain, because they're [patients] waiting for a hip replacement, but then we can divvy out and think okay, well actually there's this percentage of patients out of the 700 …, that their symptoms aren't attributed to their Heart Failure. (Specialist nurse and business manager for the VBHC team)

###### 4.2.1.1.1 Reduction of Unnecessary Appointments and Care Closer to Home

**FIGURE 4:**
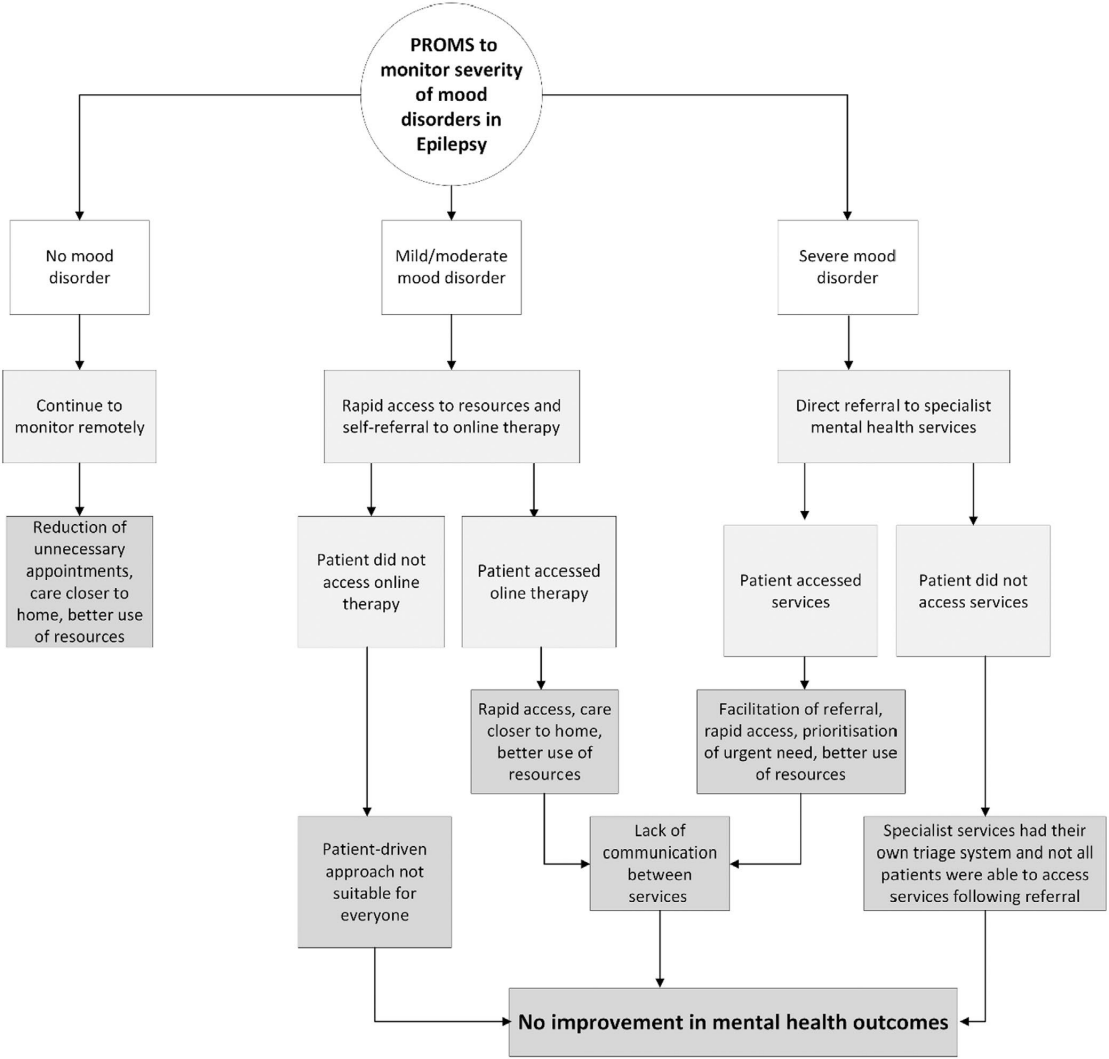
Patient‐Reported Outcome Measures (PROMs) used to monitor severity of mood disorder in Epilepsy.

In Epilepsy, nurses and multidisciplinary team members used PROMs to remotely monitor patients' mental health and tailor care pathways according to the severity of mood disorders identified (Figure 4).

We found that PROMs helped Epilepsy nurses and multi‐disciplinary team members to identify remotely those patients with no changes to their mental health, which reduced the number of unnecessary appointments. This in turn enabled better distribution of resources to those with more complex needs. Additionally, PROMs helped to monitor mood remotely as nurses believed patients were more likely to disclose mental health issues in the questionnaire rather than during a face‐to‐face appointment. Accounts from both patients and staff agreed that PROMs were useful for remote tracking and monitoring patients who were not attending clinics:

It certainly helps remotely, because we have [PROMs coordinator] doing this big cohort of people behind the scenes, who are not coming to clinic, that's been a really great help, knowing that those people [patients] have got some sort of monitoring going on (…). (Consultant—Neurologist)

However, whilst remote monitoring was well‐received by most younger patients and those with well‐controlled Epilepsy, it was not viewed positively by everyone. Most patients stressed that they preferred to have appointments face‐to‐face and expressed concerns about PROMs being used as a replacement for their appointments.

Notably, PROMs were not the only instrument used by the team to help monitor mood disorders. For example, the Epilepsy team operated an open‐access telephone helpline. Patients often called about mood issues and were then sent a text prompting them to complete a PROM. Based on the scores, appropriate actions followed, as outlined earlier. Here, PROMs assessed mood disorder severity rather than enabling remote monitoring.

###### 4.2.1.1.2 Monitoring Parkinson's Disease Patients to Help Make Evidence‐Informed Decisions About Patient Care

In Parkinson's Disease, PROMs were introduced to enable nurses to remotely monitor patients' health, improve patient stratification, optimise resources, ensure timely care, enhance service quality, and promote better quality of life, motor function, and autonomy. Our findings showed no evidence that PROMs achieved these outcomes. Questionnaire data revealed that one of two Parkinson's nurses found PROMs helpful, primarily because PROMs data were not accessible in time to inform symptom monitoring or treatment adjustments. Patients received PROMs every 6 months, either independently of their appointments or during them, resulting in outdated or unavailable information when needed. This limitation undermined the utility of PROMs in monitoring patients and achieving the intended outcomes. Nonetheless, similar to Epilepsy services, all patients had direct access to the specialist nurse, either via a helpline or email, and could make contact whenever they had concerns. These were the only two services that facilitated direct communication with clinicians.

##### 4.2.1.2 Use of PROMs to Facilitate Referral

The Heart Failure service had an efficient system in place to refer patients to other specialties which, at first, was entirely based on a clinical assessment of the patient without the use of PROMs. However, following our evaluation and feedback, the nurses decided to modify the PROMs so they could be used to help inform referrals. This ensured that the specific needs and priorities of patients were considered during the referral process as patients were able to share their treatment goals via PROMs.

###### 4.2.1.2.1 Signposting Epilepsy Patients to Mental Health Resources and Facilitating Referral

In Epilepsy, patients whose PROMs scores indicated mild to moderate mood disorders received a follow‐up paper‐based letter in the post communicating the results of their PROMs scores along with online resources and information for self‐referral to online Cognitive Behavioural Therapy (CBT) (Figure 4). In these instances, PROMs helped to facilitate rapid access to resources and self‐referral. Sixty percent (*n* = 40) of Epilepsy staff perceived that patients' symptoms and needs were more quickly identified and acted upon with the use of PROMs. However, most patients were not motivated to self‐refer to online CBT solely based on information provided in the letter. In such cases, no actions happened from completing PROMs except to inform the patient regarding their mental health issues. Patients shared an expectation that PROMs could trigger an appointment with mental health professionals to address their mood disorder. Patient's expectations of a healthcare professional‐driven rather than a patient‐driven response could be rooted in the medical model of care where the patient assumes a more passive role. But it could also be linked with the patient's mood disorder and how they generally felt about their care, as expressed in the following account from a patient:

(…) I mean how many depressed people do you know that will actually ring up and jump through hoops asking for help [from specialist mental health services] after they've been repeatedly kicked by a department. Patient interview (36–50 years, male, married, diagnosed with Epilepsy over 10 years ago)

We found no evidence that patients who self‐referred perceived online CBT as effective for treating mood disorders. Insights from PPIE suggested that these CBT sessions were aimed at the wider public and did not meet the needs of patients with Epilepsy. For instance, not only do medications for Epilepsy can impact patients' mental health, mood, and personality, but mood disorders arising from Epilepsy are often associated with profound life‐changing effects of living with the condition.

Despite this finding, PROMs helped facilitate the identification of high‐risk Epilepsy patients with severe mood disorders, and both patients and nurses agreed that PROM scores were useful as evidence of the need for urgent referral to mental health services. However, patients referred to specialist mental health services for severe mood disorders often encountered difficulties accessing treatment following their referral, as detailed in the account below:

(…) So, I've seen the letter that goes to the care team [specialist mental health service]. So, the care team are fully aware and he [Epilepsy consultant] is voicing his concerns about how high the PROMs scores are, so they know but they [specialist mental health services] don't do anything about it (…) (36–50 years, male, married, diagnosed with Epilepsy over 10 years)

Although two out of five Epilepsy staff members believed PROMs enabled quicker access to mental health support, no patients shared this view. However, interviews with nurses and multidisciplinary team members suggested that uptake of specialist mental health services was limited due to a lack of resources and long waiting lists. In addition, specialist mental health services had their own triage system and performed their own assessment for all patients. As a result, not all patients who were referred by the Epilepsy team were able to access these services. It is important to highlight that patients can also be referred to these services by their general practitioner. Some Epilepsy nurses and multidisciplinary team members had reservations about using PROMs to measure mood disorders without having any resources or staff allocated to deal with the issues within the neurology department:

Well, the reservations I already mentioned, about you know, being the person to identify a lot of mental health concerns, and risk, there are questions there about whether patients think of ending their life, and so you become the owner of that risk and that's difficult, particularly like I said, when you haven't got any resources to fix it. So, we don't have any mental health crisis services in neurology, we've got to then try and engage other services. I mean, I had reservations about creating awareness of a problem that we didn't have a solution for, within our team, or even our department. (Neurologist, interview 04 DS700097)

Of note, during the research study we had to activate a safeguarding concern about one participant with Epilepsy who needed urgent access to mental health support.

Overall, while PROM implementation in Epilepsy reduced unnecessary appointments, promoted care closer to home, and facilitated referrals, it did not help inform Epilepsy treatment adjustments or improve mood disorders—the programme's primary objectives. Additionally, the Epilepsy team could not track patients' progress or outcomes, as they lacked access to notes from mental health treatments, such as online CBT or specialist services (Table 4).

###### 4.2.1.2.2 The Use of PROMs to Facilitate Referral in Parkinson's Disease

In Parkinson's Disease, we found no evidence from patients' interviews describing that PROMs helped facilitate referrals. The lack of staff training on the use of PROMs as part of VBHC and poor adherence explained why PROMs were not used to facilitate referrals. For instance, only one specialist nurse was using PROMS at the time of interview, and described they would only look at PROMs when prompted by a patient. When asked specifically about how PROMs could help facilitate referrals, they said that PROMs did not give them enough information to help them make a referral:

I don't feel that I gained or have gained enough information from PROMs to then make the referrals. They [PROMs] would have picked up some things which is helpful, but it wouldn't have picked up all specifics which would have allowed me to write those letters of referral without a consultation. [Parkinson's Disease specialist nurse]

However, within a VBHC context, PROMs were not meant to be used for clinical assessment and diagnosis. Instead, PROMs should be used to help monitor symptoms and highlight unmet needs, so these can be discussed with the patient during a consultation leading to shared decision‐making regarding treatment and referral. The loss of cognitive and physical functioning was a major concern for patients as they would not be able to drive or do the activities of daily life as before. Recently diagnosed patients or those with milder symptoms were concerned about the deteriorating nature of the condition and wanted access to a multidisciplinary team, especially physiotherapy and occupational therapy, before their condition started deteriorating to help them maintain function and autonomy for as long as possible. However, care for these patients was mainly focused on medication and adjusting medication dosage.

##### 4.2.1.3 Triage of Patients for Cataract Surgery Based on Urgency of Need and Quality of Life

We found no evidence PROMs helped facilitate triage of patients for surgery, impacting all subsequent outcomes. Surgery referrals relied on optician reports regarding patient symptoms, visual acuity, and clinical assessments. Evidence suggested that the PROMs electronic system implemented was not fit for purpose and increased staff workload. For instance, PROMs were initially collected on paper and had to be manually inputted and calculated by a PROMs administrator, rendering their use impractical:

… a lot of patients [PROMs] still come in on paper from the opticians, but it's not being processed. And when we triage the referrals, to be honest, we tend to just ignore those bits of paper [paper version of PROMs] because the response would have to be you can't just add up the scores on the PROM score sheet, it's not as simple as that. It has to go into a spreadsheet and then each score is weighted, and so that we normally just triage based on what the optician has written in their letter. (Ophthalmologist consultant)

Even as the implementation progressed and scores became available in the IT system, the lack of integration with patient records hindered the use of PROMs for triage and this VBHC programme was discontinued. Staff and patients proposed a potential solution involving the use of a traffic light system, based on PROMs scores, to inform triage in the Cataract surgery pathway. Notably, not all patients with concerning PROMs scores would benefit from or be suitable for surgery, especially if they have other eye disorders alongside cataracts, such as glaucoma or macular degeneration, which could impact their PROMs scores. Hence, optometrists should undergo training to use PROMs assessments and scores in determining whether a patient should be referred for surgery. Additionally, integrating CROMs into the pathway could offer an additional clinical context to PROMs scores, similar to the current process used in Heart Failure.

#### 4.2.2 VBHC Intervention at Patient Level (CMO Chain 2)

Some evidence suggested PROMs helped improve awareness of symptoms in patients with Epilepsy and Heart Failure but these did not translate into better health outcomes (Figure 2).

##### 4.2.2.1 Self‐Monitoring Symptoms to Increase Awareness of Health and Improve Self‐Management and Shared Decision‐Making

We found no evidence that patients used PROMs to self‐monitor their health, which impacted all subsequent outcomes. One possible explanation was that filling out a PROM raised symptom awareness, but patients did not have access to their PROMs scores to help them track changes over time. As a result, patients perceived PROMs as something only designed to help services and healthcare professionals. Despite that, most patients acknowledged it would be useful to track and compare their scores over time to reflect on their health and symptoms and subsequent self‐management.

In addition, most patients with Epilepsy were not aware that mental health disorders could affect their Epilepsy management. Consequently, they did not perceive PROMs as part of their Epilepsy care and were not interested in using them for self‐monitoring. Despite that, some female patients with Epilepsy and Heart Failure described that completing PROMs helped them understand more about their mental and physical health, respectively.

It's good to sort of touch base with things like [living with a chronic condition]. Because you're going through this illness all the time, and you're taking your medication, and you do all what you're supposed to be doing, turn out for your appointments and things like that. But whether you'll actually sit down and think about it, you know, it's a good thing to do. And I think that that questionnaire [PROMs] helped me to do that, you know? (51–70 years, female, single, diagnosed with Heart Failure)

Empowering patients to actively self‐monitor their health with PROMs could lead to greater involvement in their treatment and self‐management. For this to happen, patients should receive feedback from PROMs, have access to their scores to follow changes over time, and have the opportunity to discuss strategies on how to deal with each change and new symptoms during appointments. This could enable patients to actively participate in decision‐making regarding their management and care. Notably, not all patients wanted to receive their PROMs scores or were interested in being actively involved in their own care. In general, older patients and those with mild symptoms or asymptomatic, preferred not to receive feedback from the questionnaire or participate in decision‐making.

##### 4.2.2.2 Self‐Monitoring Is Not Always Helpful

Cataract surgery differed from other long‐term condition management, as patients cannot independently self‐manage to improve their visual quality of life. As such, self‐monitoring would only serve to highlight symptoms and disease progression, but without intervention (i.e., surgery), and this could potentially exacerbate patients' feelings of hopelessness. For instance, patients expressed anxiety about gradually losing their eyesight, with some eventually experiencing blindness and becoming reliant on family. Older patients recounted instances of falls and subsequent loss of mobility and autonomy while waiting for surgery, permanently affecting their wellbeing and quality of life:

(…) I had to depend on my son taking me everywhere, and you know, like even shopping, like I couldn't see the labels on the cans, I couldn't see the prices. Even falling over, I know it sounds stupid, but I was falling off the kerb, I was bumping into things, and there was loads of times that I just [fell] flat on my face (…) (36–50 years, male, married, 4 years since diagnosis)

It is important to consider that the Cataract pathway was based on triaging patients for surgery, hence, it would not be appropriate for this service to address these issues. However, PROMs should be used to help inform whether patients should be fast‐tracked for surgery with patients involved in decision‐making during this process to determine whether surgery is the best option for them. In addition, PROMs could have an important role in preventing the negative impacts of sight loss as patients wait for surgery. For instance, there could be a system using PROMs scores to help refer and/or signpost patients to resources available in the community and other services.

##### 4.2.2.3 Measuring What Matters Most to Patients and Carers

PROMs did not always cover what mattered most to patients and their family members and/or carers, potentially impacting patient‐level outcomes. For instance, patients with Epilepsy prioritised finding effective medication with minimal side effects to control seizures, while patients with Parkinson's Disease prioritised maintaining motor function and autonomy. When poorly managed, these neurological conditions affected various aspects of life, including autonomy, employment, finances, and relationships, and often led to dependence on family members/carers. Long‐term patients felt that PROMs overlooked significant life events. For instance, some accounts from women described the challenges of managing Epilepsy during pregnancy or menopause and being denied certain medications during childbearing age. Similarly, patients with Parkinson's Disease found PROMs too lengthy and repetitive, missing key areas such as multidisciplinary team support earlier and medication management. Some patients also had to care for family members themselves even though they feared for their future and loss of function.

PROMs did not address medication‐related issues or safety concerns for patients living alone. In addition, PROMs were not suitable for patients with complex needs and advanced illnesses. Some of the PROM questions were not appropriate due to the advanced symptoms these patients had. These patients relied on their family members/carers due to their advanced symptoms (memory and cognitive loss, aphasia, major loss of function, etc.), and that included help with completing PROMs. Apart from concerns regarding how their relationship might impact PROM completion (with some patients hesitant to share symptoms or mental health concerns), it is crucial to recognise that family members/carers also had unaddressed health needs, which were not covered by PROMs.

##### 4.2.2.4 VBHC Intervention at Service Level (CMO Chain 3)

Service level outcomes were only observed in Heart Failure (Figure 2). We found no evidence that aggregated PROMs were being used to help inform the redistribution of resources in the other three services, which affected all subsequent outcomes. A potential explanation for this was the lack of IT integration with patient records and the lack of an easily accessible system to help staff and patients access aggregated PROMs scores over time.

In Heart Failure, we found some evidence that PROMs were used to create a better understanding of the patient population and their needs, to help tailor services and to strategically plan the distribution of resources. This resulted in a level of service redesign and increased the quality of care:

…And then what I can do is if I pull my PROM data, and really analyse that PROM data, I can see what I've got in my [case load]. So I can turn around and say to you, well, I've got 150 that need this, I've got 200 [patients] that need that, and I've got 300 [patients] that need this. So from an operational perspective, I can manage, and then I can break that down into locality, so I can see well actually, I don't need five clinics for more complex patients, in this area. I need three clinics of more rapid medication changes. Because the population from my PROMs and my data, is telling me this. So I can maximise my nursing resource better. [specialist nurse and business manager for the VBHC team]

Increasing resources in areas of higher need resulted in patients having to wait fewer days for an appointment. For instance, in the Heart Failure service, the average wait before the introduction of PROMs was around 70 days and dropped to 12 days in year one, 22 days in year two, and 17 days in year three. It is also important to highlight that PROMs were implemented alongside an electronic referral system to ensure that all referrals to the service were appropriate and that four new members of staff were hired. This significantly reduced staff caseload (i.e., from 120 patients to 50 patients per nurse), in turn allowing resources to be shifted to areas of higher need, which positively impacted outcomes such as waiting times and facilitation of rapid access for those with urgent needs.

Despite the lack of evidence to suggest that PROMs were used to inform service redesign in the other three services, we found some evidence that aggregated PROMs data helped to identify the need to change the provision of service in Cataract surgery. Comparison of PROMs scores before and after surgery across patients demonstrated the enhanced benefit of cataract surgery on the second eye and that the surgery on the first eye alone caused imbalance and resulted in worse outcomes. However, only a small percentage of patients were completing PROMs post‐operation, and we were unable to determine whether the provision of care was changed as a result. Evidence suggested that some patients still had to wait a long time to receive surgery for the second eye, as described by a carer in the account below:

About two years ago, she lost her sight completely, and was completely blind for almost a year and that greatly affected her mobility and her confidence and her ability to do anything. And even now that she's had one cataract done, so she has sight in one eye, she's very unbalanced, lopsided, you know, she's only got that vision, and it hasn't sort of brought back the confidence (> 70 years, female, widowed, mother's carer)

### 4.3 COVID‐19 Pandemic

The use of PROMs was disrupted by the COVID‐19 pandemic. Patient appointments shifted to remote formats; PROMs were switched to collection online or over the phone. By the time of the interviews during the post‐pandemic recovery period, face‐to‐face appointments were available alongside virtual options, and in the Parkinson's Disease clinic, in‐person support was available again to complete PROMs.

## 5 Discussion

This study represents the first large‐scale realist evaluation of PROMs within VBHC programmes, addressing a significant gap in rigorous evaluation within this field. The findings offer new insights across five areas: design and implementation considerations, the advantages and challenges of integrating PROMs into routine practice, and the roles of patients, healthcare professionals, and VBHC programmes in achieving desired outcomes.

Firstly, it became clear that the strategy for implementing PROMs needed to be both realistic and achievable. Consequently, the initial programme theory should rely on a straightforward chain of cause and effect/impact and remain within the influence of committed and motivated clinicians.

The one VBHC programme where PROMs were seen to make the most difference (Heart Failure) had an inspirational and motivated nurse leader, and the wider team saw the potential benefits and rewards of using PROMs to triage the right patients to the most appropriate care pathway for which the Heart Failure nursing team was responsible for delivering. They used PROMs to monitor symptoms over time, thereby increasing staff awareness, optimising resources, reducing waiting times, and improving health outcomes. Within the four programmes we evaluated, Heart Failure was the only service that used aggregated PROMs scores to identify areas of higher needs across the service for resource management and healthcare delivery. PROMs demonstrated substantial social and economic return on investment and real transformation in Heart Failure services. In our SROI reported elsewhere, the Heart Failure service generated £5.55 of value for patients and the NHS for every £1 invested (Crane et al. 2024).

Second, the reliance on complex, lengthy, and standardised outcome measures, originally developed for use in trials, requires reconsideration. In some cases, these measures may exacerbate inequity rather than reduce it. In trials, patients are typically required to complete PROMs only at a few key time points before discontinuing. It is well known that completion rates for outcome questionnaires decline as trials progress. Despite this, there is an assumption that these structured outcome questionnaires can be seamlessly adapted for routine use, even over a lifetime, in the context of long‐term conditions. Our findings indicate that this is not the case. The PROMs used in each service were long, only available in English, repetitive, and generally did not consistently focus on what patients valued and what clinicians needed to inform shared decision‐making. These PROMs often failed to accommodate the specific accessibility needs of the patients expected to complete them. This included patients with conditions such as Parkinson's and Epilepsy, who may experience memory difficulties, as well as those who lacked the capacity to complete them, were not digitally literate, or did not have English as their first language. None of the questionnaires followed patient‐facing design principles using patient information standards and best practice principles. If we had set out to co‐design a set of new resources for patients to use in routine practice for this purpose, it is highly unlikely that they would look anything like PROMs used in research studies and trials. Although nurses attempted to simplify and shorten some questionnaires and provided nursing assistants to support completion, these efforts did not fully mitigate the risk of increasing inequity rather than reducing it when introducing PROMs into routine care. Notably, while adapting PROMs to better fit routine care improved compliance, it invalidated the questionnaires, hindering comparisons across services and organisations (Juniper 2009).

Overall, apart from the Heart Failure service, it was unclear what benefit PROMs had over data collected routinely by clinicians, or whether using a standard shared decision‐making model prompting patients to ask their clinicians three questions during their consultation would be more patient‐friendly and acceptable to patients and carers. See, for example, the shared decision‐making model in Supporting Information S9 ‐ Shared Decision‐Making Model. Of note, in the post‐pandemic period, the ABUHB sent out a text with three questions to all patients who had their treatment delayed or halted in order to triage those with the greatest need and received an 80% response rate, thereby indicating that less is often more.

Third, PROMs role in facilitating patient participation as equal partners in their own care was minimal, if not non‐existent. The message patients received indicated that they needed to complete a PROM for their clinical team. There was little, if any, patient education about how they could use PROMs to help their self‐management of long‐term conditions. Patients generally did not receive feedback on their scores, could not access their completed PROMs after submission, and had no way to track changes over time due to the lack of a patient‐facing dashboard. Consequently, they were unable to use PROMs for self‐monitoring, which limited their ability to achieve better self‐management, enhanced communication, and shared decision‐making. Empowering patients to actively self‐monitor their health with PROMs could lead to greater involvement in their treatment and self‐management. For this to happen, patients should receive feedback from PROMs, have access to their scores to follow changes over time, and have the opportunity to discuss strategies on how to deal with each change and new symptoms during appointments. This could enable patients to actively participate in decision‐making regarding their management and care.

While PROMs helped patients voice concerns during consultations (Greenhalgh et al. 2013), and provided a structure for raising sensitive issues, such as mental health disorders, they did not provide a framework for changing clinicians' communication practices (Greenhalgh et al. 2017). Other studies also raise concerns regarding the level of standardisation introduced by PROMs and whether it could be harmful to patient‐clinician relationships, communication, and shared decision‐making (Mitcheson and Cowley 2003; Rhodes et al. 2006). Despite that, we found that PROMs increased health awareness in conditions such as Heart Failure and Epilepsy, but this alone was insufficient to elicit any further outcomes. Increased awareness of health can only result in improved self‐management and other positive outcomes if patients possess the necessary knowledge, skills, confidence, and are actively involved in communication (Field et al. 2019). Collaboration between patients and clinicians is essential, wherein clinicians offer guidance and strategies to empower patients to assume control of their health. Increased patient involvement in their own care not only improves clinician management and shared decision‐making but also enhances patient self‐management (McGreevey 2006). Support is also needed for clinicians to involve patients in decision‐making. Pressured by time constraints, clinicians often rely on their expertise to make decisions, perceiving it as quicker and easier. Yet, engaging patients in their care can save time in the long run by enabling them to better manage their conditions and recognise when to seek help. However, achieving this warrants a shift in culture from the medical model to a person‐centred care at all levels, including clinicians, patients and organisations (van Engen et al. 2021). This helps explain why many patients saw the potential benefits of PROMs but felt that they did not actually get any benefit from completing PROMs. Completion rates were low, as patients often saw little value in completing them, especially when it was unclear how clinicians used the information.

Fourth, although Wales has a national VBHC programme and enthusiasm for implementing PROMs at a programme level, support for their local use in routine clinical services has waned over time, except in the nurse‐led [with consultant oversight Heart Failure service]. Our evaluation found that implementing PROMs is resource‐intensive and challenging, with barriers similar to those identified in our scoping review (Silveira Bianchim et al. 2023). Implementation failure in particular happened when PROMs were not fully integrated into existing IT systems. Time‐pressed consultants and nurses disengaged from using PROMs when it required additional time and effort to access PROMs data. In most cases, PROMs collection and use heavily relied on nurses, as technical challenges hindered consultants' ability to visualise and use cumulative data to track trends. For example, PROMs collection ceased in Cataract surgery due to technical and access issues, and in Epilepsy, only one of the Health Board's services used PROMs. In addition, PROMs data collected for Parkinson's Disease patients were not being used or shared across services, despite their diverse needs and co‐morbidities requiring coordinated care. This raises ethical concerns, as it is not justifiable to ask patients to invest time and effort in completing PROMs if data are not used to inform their care or improve services. Patients with complex conditions, such as those with palliative care needs or cognitive impairment, might benefit more from individual tailoring of treatment and integration of PROMs use across different services (Greenhalgh et al. 2017). The use of PROMs in managing complex patients and co‐morbid conditions is often hindered by several barriers, including a lack of integrated clinical leadership and PROMs use across relevant services, delays in data availability and sharing, and unclear responsibilities due to inadequate planning and coordination (Benson 2023). Additional challenges include inadequate infrastructure, concerns about misuse of results for cost containment or eligibility decisions, measurement complexities, and IT limitations. Similar challenges are observed in other complex conditions, such as cancer, where time constraints and limited expertise among health professionals prevent the effective integration of PROM data into clinical practice, thus limiting its practical use (Nguyen et al. 2021). Findings suggest that increasing referrals and prioritising access based on urgency alone is insufficient to improve outcomes without robust systems to address the issues identified by PROMs. Thorough implementation planning is crucial to define responsibilities and navigate the complexities of healthcare systems and conditions.

## 6 Strengths and Limitations

Realist Evaluation is a robust theory‐based methodology that allows a comprehensive assessment of complex interventions such as PROMs within VBHC programmes. Our approach involved using diverse qualitative and quantitative data sources, encompassing 105 realist interviews conducted with patients, staff, and family member carers across the four services. We acknowledge that while the sample was limited in diversity, it was representative of the population of Wales, where the study was conducted. PPIE input was also invaluable in addressing gaps in evidence and interpreting data during the analysis of the scoping review, as well as in the development and testing of the programme theories. PPIE provided crucial insights during the development of patient questionnaires and ensured that our findings were tailored into appropriate and relevant recommendations.

Permission was obtained to verify whether patients had completed PROMs (which they mostly had), and we also observed consistency across all interviews. Response rates were also negatively affected by the high numbers of people declining to complete PROMs, as well as those who could not remember completing PROMs. Of note, we have been entirely transparent about recruitment and response rates, which is not common in other published realist evaluations.

In Table 5, we outline key recommendations to further optimise implementation and use of VBHC programmes using PROMs.

**TABLE 5:** Recommendations for the implementation and use of Patient‐Reported Outcome Measures (PROMs) as part of Value‐Based Health Care (VBHC) Programmes in long‐term and acute care.

Main recommendation	Description
Redesigning PROM questionnaires/resources for routine care	Redesign PROMs to feature shorter questionnaires with a user‐friendly format, incorporating visuals and language devoid of clinical jargon. Ensure PROMs encompass outcomes crucial to patients. For example, Epilepsy PROMs could encompass inquiries about memory loss, cognition, medication side effects, seizure types, and sleep quality. Additionally, consider incorporating a brief free‐text option to capture patient‐important outcomes not covered in the standard questionnaires. PROMs redesign should be co‐produced with relevant stakeholders
Using, managing and sharing PROM scores	Patients should receive feedback on their PROMs scores, and any changes should be discussed to aid shared decision‐making, enhance communication, and ultimately improve outcomes. In conditions like Parkinson's Disease, discussions about score changes could focus on maintaining function and autonomy, adapting to new symptoms, or managing emerging ones. Similarly, in Epilepsy, changes in scores might prompt medication adjustments, specialist referrals, or revisiting the care pathway. For Heart Failure, PROMs discussions could help determine suitability for specific treatments or identify the need for referrals. A patient‐level dashboard should be available for those interested in monitoring their scores over time to support self‐management. Careful consideration is also needed when signposting patients with moderate or severe mood disorders to self‐refer to mental health services, and family members or carers should be involved where appropriate to facilitate this process. Additionally, anonymised data from PROMs should be made available to research institutes and universities to further the understanding of surgical treatments and the management of long‐term conditions
Additional resources, training and education	Patients and staff should receive training and resources on the use of PROMs as part of routine care within a VBHC context and VBHC values. Patients and carers should receive enough resources and education on how and why to use PROMs as part of their routine care. For instance, PROMs could contain a QR code with information about the condition and VBHC. Alternatively, paper and electronic information resources could be distributed
Service Integration	PROMs should be used to mitigate service fragmentation by prioritising patients' needs and improving integration with other services and resources, such as third‐sector organisations, social services, and social care. For instance, high‐risk patients—those with severe mood disorders, complete loss of function, or a risk of falling—could benefit from better links to these additional services. Similarly, patients awaiting or unsuitable for Cataract surgery could be referred to support services to help them manage vision loss more effectively
IT support and automation	Integration of IT with the existing system is vital. Dedicated IT personnel should be accessible to assist services with the implementation of the new online system, while current staff members should undergo training to operate it proficiently. Automation of certain processes should be considered where appropriate to alleviate staff workload
Using PROMs to triage based on urgency (Cataract surgery)	PROMs completion should be done during referral or while patients are waiting to have their first appointment to help triage patients and prioritise urgency of need (Cataract Surgery context)

## 7 Conclusions

This study highlights the need for a more critical and context‐specific approach to implementing PROMs in VBHC. For PROMs to have a meaningful impact on care, programmes must move beyond generic implementation and embed them within responsive systems that consider the realities of frontline practice. Policy‐makers and service leads should prioritise co‐design approaches that involve patients and professionals in selecting and adapting outcome measures that are feasible, relevant, and actionable. Future work should focus on testing co‐developed tools, strengthening digital infrastructure, and evaluating longer‐term effects on patient outcomes and system efficiency across diverse clinical pathways.

## Disclosure

*Statements*: The authors have checked to make sure that our submission conforms as applicable to the Journal's statistical guidelines described.

## Ethics Statement

The study was approved by Wales Research Ethics Committee #5 (22.WA/0044).

## Consent

We confirm that all participants provided written informed consent before taking part in the study.

## Conflicts of Interest

Jane Noyes is Editor of the Journal of Advanced Nursing and was not involved in the editorial processing of this manuscript. All other authors declare no conflicts of interest.

## Supporting information


Data S1.

Data S2.

Data S3.

Data S4.

Data S5.

Data S6.

Data S7.

Data S8.

Data S9.

## Data Availability

All data is already reported in this paper.
