# Supplementary material for: A Realist Evaluation of the Implementation and Use of Patient‐Reported Outcomes in Four Value‐Based Healthcare Programmes
Source: J Adv Nurs. 2025 Jul 28;82(4):3678–701. doi: 10.1111/jan.70018 (PMC12994664; doi:10.1111/jan.70018)
Supplement: Supplementary file 1 — Data S1. [file JAN-82-3678-s005.docx]

**Supporting Information S1 – Environment surrounding the evaluation**

The Aneurin Bevan University Health Board (ABUHB) serves a population of 600,000 people in Wales and has integrated PROMs into 25 disease areas as part of a comprehensive VBHC programme. PROMs were collected through a hybrid approach, allowing completion over the phone, on paper, or via digital platforms. We evaluated four VBHC programmes, including the services of Cataract surgery, Epilepsy, Heart Failure, and Parkinson’s Disease, where PROMs have been routinely collected since 2018/2019. Realist evaluation of the four services was appropriate to address the overall research aims and manageable enough to achieve the objectives within the budget and time constraints.

**Table S1** Description of the overall VBHC Programme in the adopter Health Board, and the services

| **Initiative** | **Patient population** | **Description** |
| --- | --- | --- |
| First adopter Health Board (Aneurin Bevan UHB) implements VBHC from 2015 to present (2024). 26 disease conditions included to date. | The Health Board serves approximately. 639,000 patients: approx. 21% of the total Welsh | VBHC: puts a focus on the value of outcomes achieved for patients. Value in health care is realised when we achieve the best possible health outcomes for our population within the |
| **Services** | **PROMs completion** | **PROMs were used to** |
| Heart Failure routine collection of PROMs 2018 to present | 927 PROMs completed with an average completion rate of 85% | Reorganise the service more efficiently to reduce nurses’ caseloads, reduce waiting times for patients, and to focus treatment on patients’ needs |
| Epilepsy routine collection of PROMs 2017 to present | 588 PROMs completed with an average completion rate of 51% | Identify, quantify, and manage the burden of mood disorder. Identify the relationship between the patient’s mood, seizure frequency, and medication to inform future treatment options |
| Parkinson’s Disease routine collection of PROMs 2017 to present. | 840 PROMs completed with an average completion rate of 42% | Help deliver a more efficient and effective service based on disease severity and patient needs. |
| Cataract routine collection of PROMs 2017 to present | 3600 PROMs completed with an average completion rate of 88% | To redesign the referral and triage process, free up capacity and improve outcomes by treating according to need rather than waiting times. |

Key: PROMs – Patient Reported Outcome Measures; VBHC – Value-Based Health Care

# Heart Failure Service

Heart failure is a long-term, deteriorating condition. Although Heart Failure cannot usually be cured, the symptoms can often be controlled for many years. PROMs were introduced in the Heart Failure service in 2018 because the service was overwhelmed with patients and unable to meet demand. Patients were seen in an untimely manner and not necessarily in relation to their symptoms. The service introduced PROMs in an attempt to be more patient-focused. Previously, PROMs were applied at one hospital site using non-clinical staff to administer them, but for a variety of reasons, this was not sustainable.

The Heart Failure service is a nurse-led team within ABUHB providing care primarily for patients diagnosed with Heart Failure with Reduced Ejection Fraction (HFrEF). Patients with HFrEF comprised 11.3% of the total population within ABUHB (in 2019). PROMs were implemented in the service in 2018/2019 with the main priorities of ensuring that those with more urgent needs could access the service rapidly to prevent deterioration and avoidable hospital admissions and to ensure timely optimisation of evidence-based treatment to prevent mortality and morbidity. Nurses in the service have an active caseload of around 60%. In 2018-2019, the service received 556 referrals and had 208 discharges. These numbers increased to 827 referrals and 289 discharges in 2019-2020.

**Intervention**

Since initial implementation, there have been several evolutions of the care pathway to accommodate COVID-19 (see process maps in Supplementary file 2). Patients were referred to the Heart Failure service by Electronic Referral (GP/Cardiologist) or via the inpatient service (Cardiology Wards). A block booking system for appointments was also implemented to allow prompt optimisation of medication. New patients have a baseline PROMs [P1] and a **Clinical Reported Outcome Measures 1** (CROMs) [C1] completedduring an initial phone consultation with a nurse [PROMs can be undertaken via phone or on an online platform]. Information gathered is used to tailor the type and length of appointments to each patient according to their needs i.e. patients with fewer symptoms were directed to a 10-minute rapid clinic [i.e. optimisation programme] and frailer patients with more symptoms were directed to a complex clinic 30/40 minutes [i.e. the palliative/complex pathway]. Two weeks after the initial consultation, patients had a face-to-face appointment or a phone consultation where they received a second PROMs and another CROMs [quality of life P2 and a C2]. Three months after that, patients received another PROM and CROM [C3 and P3] [usually happens after 6 months from the initial appointment].

Patients who were clinically well for a whole month were discharged or referred to a Standard Clinic (for up to 12 months). All patients received a PROMs and another CROM when they were discharged [which sometimes happened before the 6-month mark] and a Patient Reported Experience Measure (PREM) after discharge (Figure S1.1). At discharge, patients followed different pathways depending on their condition [refer to logic models in Supplementary File 5]:

- - Patients self-managed their symptoms if their condition was stable and were re-referred to Heart Failure clinic if their condition deteriorated.
  - Referred to Community Care / Palliative Care [communication with Heart Failure team if needed]
  - Referred for Device Therapy [implantable electronic devices]


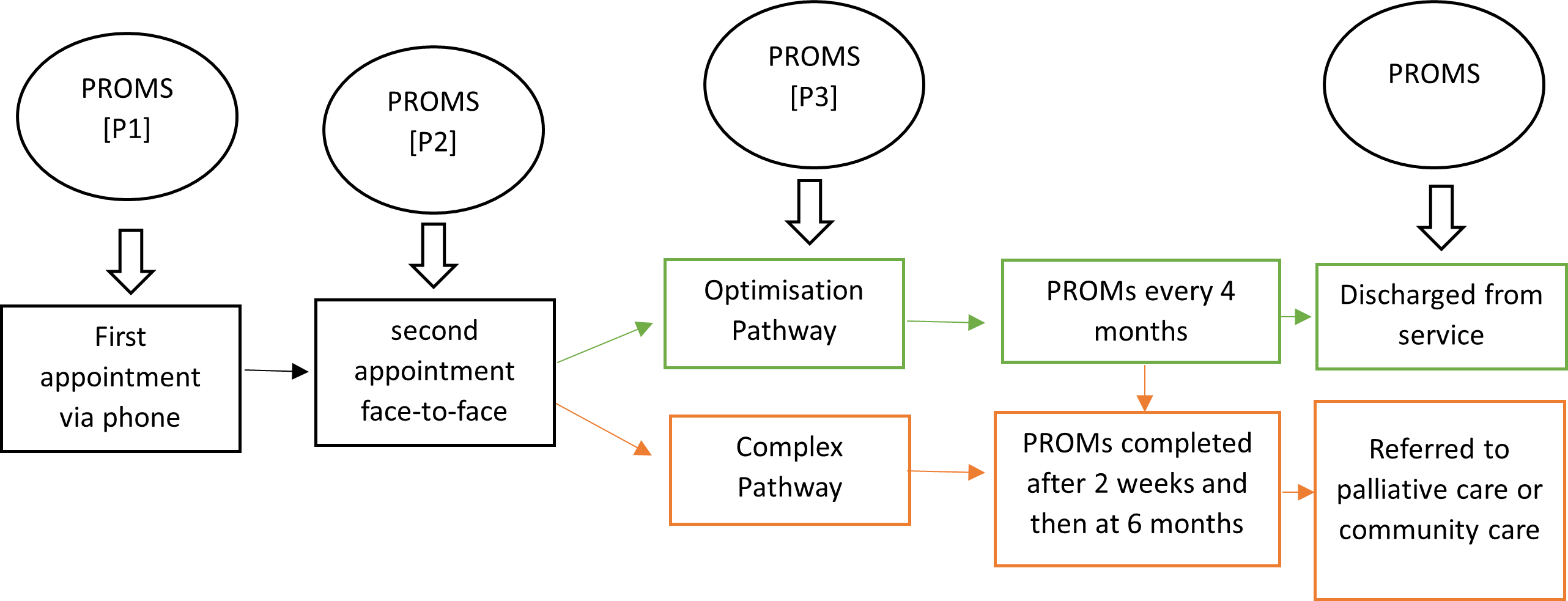


Figure S1.1 Patient Reported Outcome Measures (PROMs) pathways in Heart Failure

# Epilepsy Service

The adult Epilepsy Service in ABUHB serves a population of over 6,000 people with Epilepsy and provides an open-access service so that patients with this unpredictable condition can receive timely and responsive care. VBHC was implemented in the Epilepsy service in 2018/2019 with the aim of using PROMs to better identify and quantify the extent of anxiety and depression in people with Epilepsy. Additionally, the team wanted to improve the management of mental health at the individual patient level. The implementation of PROMs as part of routine care also envisaged achieving a data-driven evaluation of outcomes and costs to improve efficiency and effectiveness regarding the relationship between patient mood and medication and to inform treatment options. The Epilepsy PROM comprises questions on seizure frequency, plus mood scores, and a patient global impression change.

**Intervention**

The COVID-19 pandemic forced the service to move to remote consultation in up to 95% of cases. COVID-19 was also associated with an increasing demand for Epilepsy services. Since the initial implementation, there have been several evolutions of the care pathway to accommodate COVID-19 (see process maps in Supplementary File 2). The following describes the current care pathway in the post-pandemic recovery period. New patients received a baseline PROM when they first engaged with services. Patients that were already in the system received PROMs as part of their Epilepsy care via two distinct pathways – (1) ad hoc – when staff identified a need to send PROMs to patients i.e. due to concerns to their mental health; (2) a PROMs coordinator sent PROMs to patients before appointments (Figure S1.2). Subsequently, the PROMs coordinator assessed all PROMs scores which could lead to the following: (1) no action if no changes in mood were observed – in this case, patients received a letter explaining that no concerns were identified with PROMs and they would continue to receive PROMs; (2) patients identified with mild/moderate mood disorders received a letter signposting to resources and instructions to self-referral to CBT online [Silver Cloud]; (3) patients identified with severe mood disorders were referred to Community Mental Health Team (CMHT) [refer to logic models in Supplementary File 5].


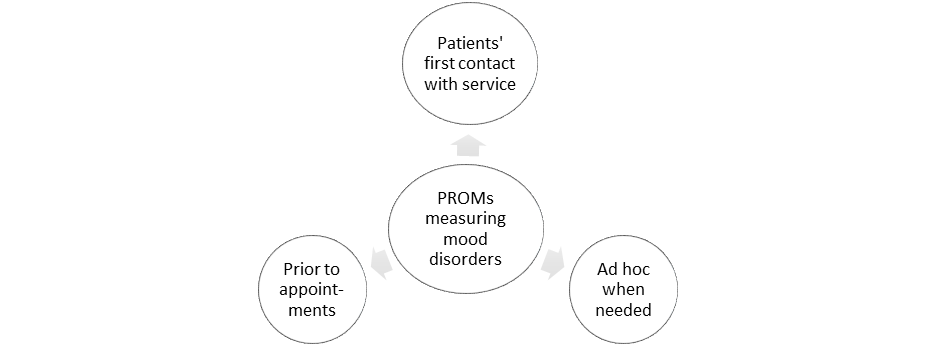


Figure S1.2 Patient Reported Outcome Measures (PROMs) pathways in Epilepsy

# Parkinson’s Disease Service

Parkinson's Disease is a progressive neurological disorder that primarily affects movement. Tremors are common, but the disorder also commonly causes stiffness or slowed movement. Parkinson’s Disease clinics are nurse-led, and the first to test the implementation framework in 2016 to help inform the expansion of PROMs to other clinical areas. The key priority of implementing PROMs was to use outcomes to deliver an efficient and effective service based on patient symptoms.

**Intervention**

The care pathway has evolved since its implementation to accommodate COVID-19 (see process maps in Supplementary file 2). However, at the time of the interviews, implementation still followed the plan created during COVID-19. Specifically, patients received PROMs during their first contact with the service, and at every six months (Figure S1.3). PROMs were also completed during face-to-face clinical appointments. A Healthcare Assistant was responsible for helping patients complete PROMs, either during clinical appointments or over the phone. Some patients also completed PROMs online from home, either independently or with help from family members [refer to Parkinson’s Disease logic models – Supplementary File 5].


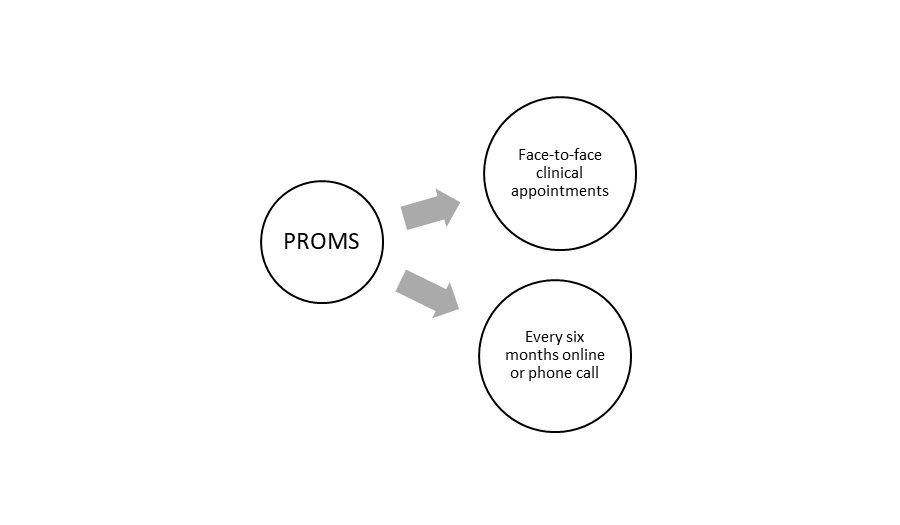


Figure S1.3 Patient Reported Outcome Measures (PROMs) pathways in Parkinson’s Disease

# Cataract Surgery Service

Cataract surgery is the most common operation performed in the UK, with more than 300,000 procedures carried out each year. Cataract surgery aims to improve quality of vision and the ability to undertake everyday tasks. ABUHB receives 6,000 referrals for cataract surgery and performs more than 3,000 cataract operations every year. Demand for cataract surgery has exceeded capacity. Within ABUHB, a pilot study identified potential variations in pathways and outcomes. The VBHC team worked with the ophthalmology directorate to set up a systematic collection of both clinical outcomes and PROMs. Visual acuity (ability to read letters of decreasing size) from a set distance was used as the clinical outcome. A cataract questionnaire consisting of nine questions (CatQuest-9SF tool) was used as the validated PROM. The Catquest questionnaire explores visual impairment from a patient’s perspective by asking questions about the ability to recognise faces, read newspapers, or undertake hobbies.

PROMs were implemented in the Cataract surgery service in 2018 (See process maps – Supplementary File 2). The intervention was interrupted during the COVID-19 pandemic in 2019, two years after implementation, and recommenced around late 2021 for a small sample of patients before withdrawing completely. The service is clinician-led and has a large caseload. The key anticipated/intended outcomes from the implementation of PROMs was to offer patients who do not need surgery alternative pathways, reduce costs, improve capacity and flow, and use outcome measures to redesign services.

**Intervention**

Before being completely withdrawn, PROMs were collected at four distinct time points: during referral at the optometrist, whilst patients waited for surgery, and pre- and post-surgery (Figure S1.4). A PROMs coordinator was responsible for manually triggering PROMs for patients on the waiting list. The coordinator also manually added patients to the PROMs pathway. Once in the pathway, patients would automatically receive a pre-operation PROM and a post-operation PROM after 8 weeks in the pathway [refer to Cataract surgery logic models Supplementary File 5].


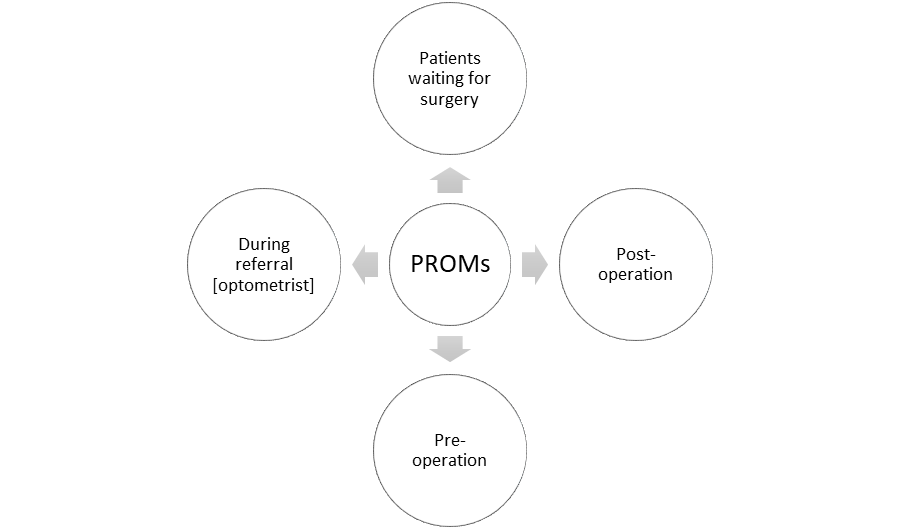


Figure S1.4 Patient Reported Outcome Measures (PROMs) pathways in Cataract Surgery
