# Supplementary material for: A Realist Evaluation of the Implementation and Use of Patient‐Reported Outcomes in Four Value‐Based Healthcare Programmes
Source: J Adv Nurs. 2025 Jul 28;82(4):3678–701. doi: 10.1111/jan.70018 (PMC12994664; doi:10.1111/jan.70018)
Supplement: Supplementary file 2 — Data S2. [file JAN-82-3678-s008.docx]

**Supporting Information 2 – Process Maps**

**
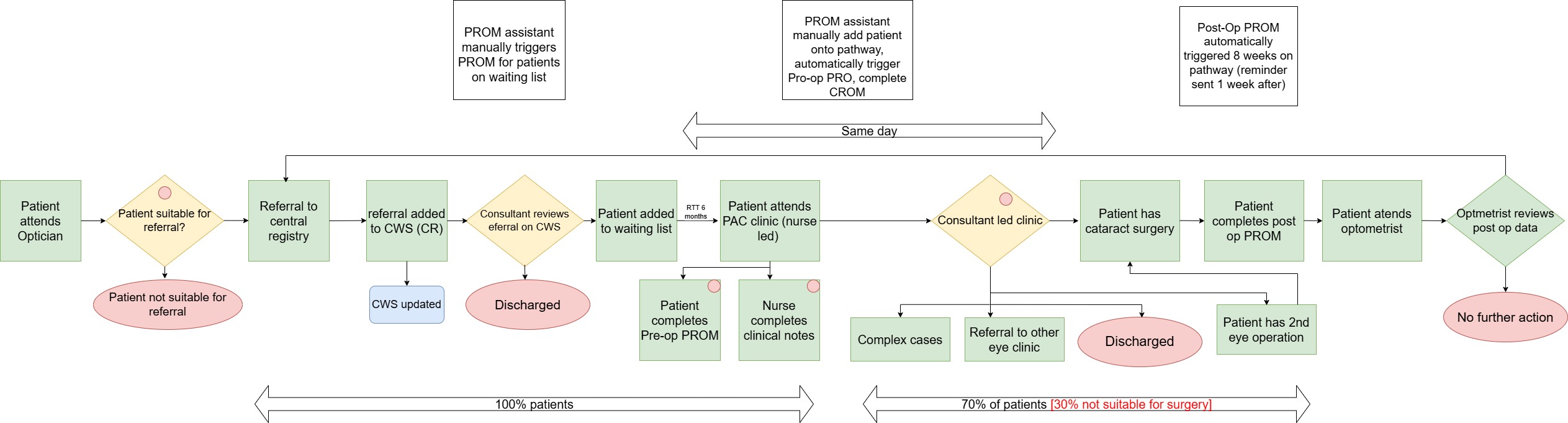
**

Figure S2.1 Process Maps – Cataract Surgery

Key: PROMs – Patient Reported Outcome Measures; CROM – Clinical Reported Outcome Measures; CWS – Clinical Work Station; RTT – referral to treatment; PAC – pre-assessment clinic; Post Op – post-operative; Pre-Op – pre-operative

**Maps**

Various referral points

Outpatient booking through central registry

4-12 weeks

Face-to-face Clinic OPA (Outpatient Appointment)

(45 minutes)

2-8 weeks

Baseline PROM (demographic): nurse looks at PROM in consultation

FU/Follow-up Outpatient Appointment

(30 minutes)

QoL PROM

(Quality of Life): nurse looks at PROM in consultation

Stable?

Yes

Echo?

No

No

Yes

**Echocardiogram**

Radiology

(30-45 minutes)

**Discharge**

(to appropriate service)

**PROMs completion 2018-2020:**

- 1 clinic, 1 patient, 1 appointment, 1 PROM

- PROMs completed either in clinic with volunteers or via online platform (Dr Doctor)

Discharge

QoL PROM

QoL PROM

Key:

**Process Step**

(Patient present)

PROM completed

Process Step

(Patient present)

Clinical Decisionn

Figure S2.2 Pre-pandemic Process Maps – Heart Failure

Key: PROMs – Patient Reported Outcome Measures; QoL - Quality of Life; Echo – Echocardiogram

Various referral points

Outpatient booking through central registry: Referral /discharge criteria modified

4-12 weeks

Telephone/Video clinic + urgent Face-to-face clinic

(45 minutes)

2-8 weeks

Baseline PROM (demographic): nurse looks at PROM in consultation

Telephone/Video clinic

FU/Follow-up Outpatient Appointment

(30 minutes)

QoL PROM

(Quality of Life): nurse looks at PROM in consultation

Stable?

Yes

Echo? **delayed**

delayeddel

No

No

Yes

**Echocardiogram**

Radiology

(30-45 minutes): **delayed during pandemic**

**Discharge**

(to appropriate service)

**PROMs completion from March -Oct 2020:**

- collected every 6 months

- PROMs completed on online platform (Dr Doctor)

- pathway as remote as possible

Discharge

QoL PROM

QoL PROM

Urgent Face-to-face clinic: clinical decision made following a telephone nurse appointment

(45 minutes)

Key:

**Process Step**

(Patient present)

PROM completed

Process Step

(Patient present)

Clinical Decisionn

Figure S2.3 Pandemic Process Maps – Heart Failure

Key: PROMs – Patient Reported Outcome Measures; QoL – Quality of Life; Echo – Echocardiogram

Electronic Referral

(GP/Cardiologist)

Back to Referrer with a letter

No

Yes

**New Outpatient**

**Appointment -PHONE**

(45 minutes)

**FU Outpatient**

**Appointment**

(30 minutes)

Telephone/face-to-face

Optimisation Patients

(4-month Programme)

**Palliative/Complex Patients**

**FU Outpatient**

**Appointment**

(30 minutes)

Repeat as needed average of monthly

QoL PROM/CROM as per ICHOM time line – 2 weeks and then 6 monthly

**FU Outpatient**

**Appointment**

(30 minutes)

**Group Consultation**

**Currently on hold**

(2 hours)

8 OPA in 4-months

Patient Optimised

**Echocardiogram**

Radiology

(30-40 minutes)

QoL PROM/CROM

2 weeks from referral date

**Discharge**

(to appropriate service)

Yes

No

QoL PROM/CROM

PREM

If patient becomes unstable, they will be removed from Optimisation and referred to the Palliative/Complex FU Clinic. Will still complete 4-monthly PROM / CROM

Inpatient service

(Cardiology Wards)

(from Sept 2022)

**Post Discharge Clinic** (30 Min)

Face-to-face or telephone

 Ward Discharge Community Hub

Baseline PROM/CROM

*→ From October 2020 PROMs collection followed ICHOM timeline (every 6 months)*

Key

QoL PROM/CROM

**Process Step**

(Patient present)

PROMs completion from Oct 2020:

- collected every 6 months

- PROMs completed on online platform (Dr Doctor) or on telephone with nurse

Clinical Decision

Process Step

(Patient present)

Figure S2.4 Post-pandemic Process Maps – Heart Failure

Key: PROMs – Patient Reported Outcome Measures; CROM – Clinical Reported Outcome Measures; GP – General Practitioner; FU – follow-up; QoL – Quality of Life; ICHOM - International Consortium for Health Outcomes Measurement; OPA – Outpatient Appointment

Patient having difficulty, e.g. increased seizures, low mood, can’t sleep, ‘unwell’, balance problems

Presents at A&E department or GP appointment

Majority of patients call Open Access answerphone service dealt with by Epilepsy Nurse Specialist (ENS)

If patient needs change in medication, referral to Neurology consultant

Booking Process

Patient receives an appointment and presents at Outpatient department (OPD)

Reception mark patient as arrived

HCSW calls patient through for Observation

Observation taken and notes put on trolley outside consultant room

Consultant reads notes and calls patient through

Consultant takes history and examines patient

PROMs completed in clinic with tablet/paper/

HCA or digitally via online platform (Dr Doctor)

Clinician who is doing the clinic looks at PROMs

Majority of calls handled and closed by ESN (no PROMs at this stage)

Figure S2.5 Pre-pandemic PROMs process map – Epilepsy

Key: PROMs – Patient Reported Outcome Measures; GP – General Practitioner; A&E – Accident and emergency; HCA – Healthcare assistant; - HCSW - healthcare support worker

Presents at A&E department or GP appointment

Majority of patients call Open Access answerphone service dealt with by Epilepsy Nurse Specialist (ENS)

Referral to Neurology consultant if medication needs to be changed

Booking Process

Patient receives an appointment and presents via telephone or video link

Consultant reads notes/looks at PROMs and calls patient

Consultant takes history and examines patient

PROMs completed digitally via online platform (Dr Doctor) or via phone

PROMs coordinator reviews PROMs scores as well

Patient with mild mental health problems is sent advice letter and added to watchlist

Patient with severe mental health problems receives further review and advice by consultant or epilepsy team refers to GP/Mental Health Team

Review with PROMs after 4-6 months and/or Referral to online CBT therapy programme

Patient having difficulty, e.g. increased seizures, low mood, can’t sleep, ‘unwell’, balance problems

Majority of calls handled and closed by ESN (no PROMs at this stage)

Review with PROMs after 3-4 months

Figure S2.6 Pandemic PROMs process map – Epilepsy

Key: PROMs – Patient Reported Outcome Measures; GP – General Practitioner; A&E – Accident and emergency; CBT - Cognitive Behavioural Therapy

Presents at A&E department or GP appointment

Majority of patients call Open Access answer phone service dealt with by Epilepsy Nurse Specialist (ENS)

Referral to Neurology consultant if medication needs to be changed

Booking Process

Patient receives an appointment and presents via telephone, video link or face-to-face

Consultant takes history and examines patient

Face-to-face: Patient receives an appointment and presents at Outpatient department (OPD)

Reception mark patient as arrived

HCSW calls patient through for Observation

Observation taken and notes put on trolley outside consultant room

Consultant reads notes and calls patient through

Patient having difficulty, e.g. increased seizures, low mood, can’t sleep, ‘unwell’, balance problems

Majority of calls handled and closed by ESN (no PROMs at this stage)

Via video link/phone: Consultant reads notes/looks at PROMs and calls patient

PROMs completed digitally via online platform (Dr Doctor) or via phone

PROMs coordinator reviews PROMs scores as well

Patient with mild mental health problems is sent advice letter and added to watchlist

Patient with severe mental health problems receives further review and advice by consultant or epilepsy team refers to GP/Mental Health Team

Review with PROMs after 4-6 months and/or Referral to online CBT therapy programme

Review with PROMs after 3-4 months

Figure S2.7 Post-pandemic PROMs process map – Epilepsy

Key: PROMs – Patient Reported Outcome Measures; GP – General Practitioner; A&E – Accident and emergency; CBT - Cognitive Behavioural Therapy

GP/other refer patient to epilepsy Outpatient Department (OPD)

Consultant reviews referral + accepts on to epilepsy clinic waiting list

Appointment booked

In days leading up to appointment, the online platform (Dr Doctor) automatically sends PROMs to all patients in the clinic

PROMs are reviewed in clinic as patients seen

Discharged

Follow up

Process will repeat PROM at the follow up appointment

Figure S2.8 Outpatient PROMs process map (pre-, post- and during pandemic) – Epilepsy

Key: PROMs – Patient Reported Outcome Measures; GP – General Practitioner


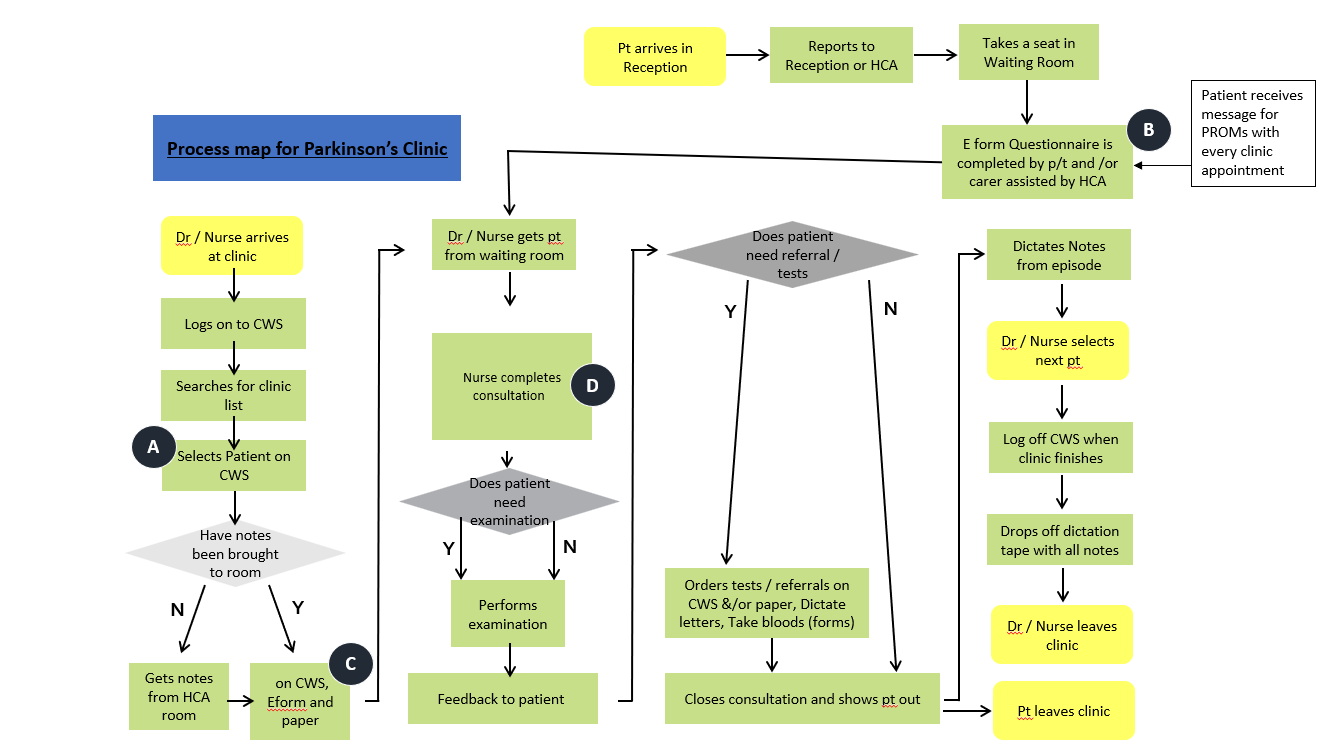


Figure S2.9 Pre-pandemic Process Maps – Parkinson’s disease

Key: PROMs – Patient Reported Outcome Measures; HCA – Healthcare assistant; pt – patient; CWS – Clinical Work Station


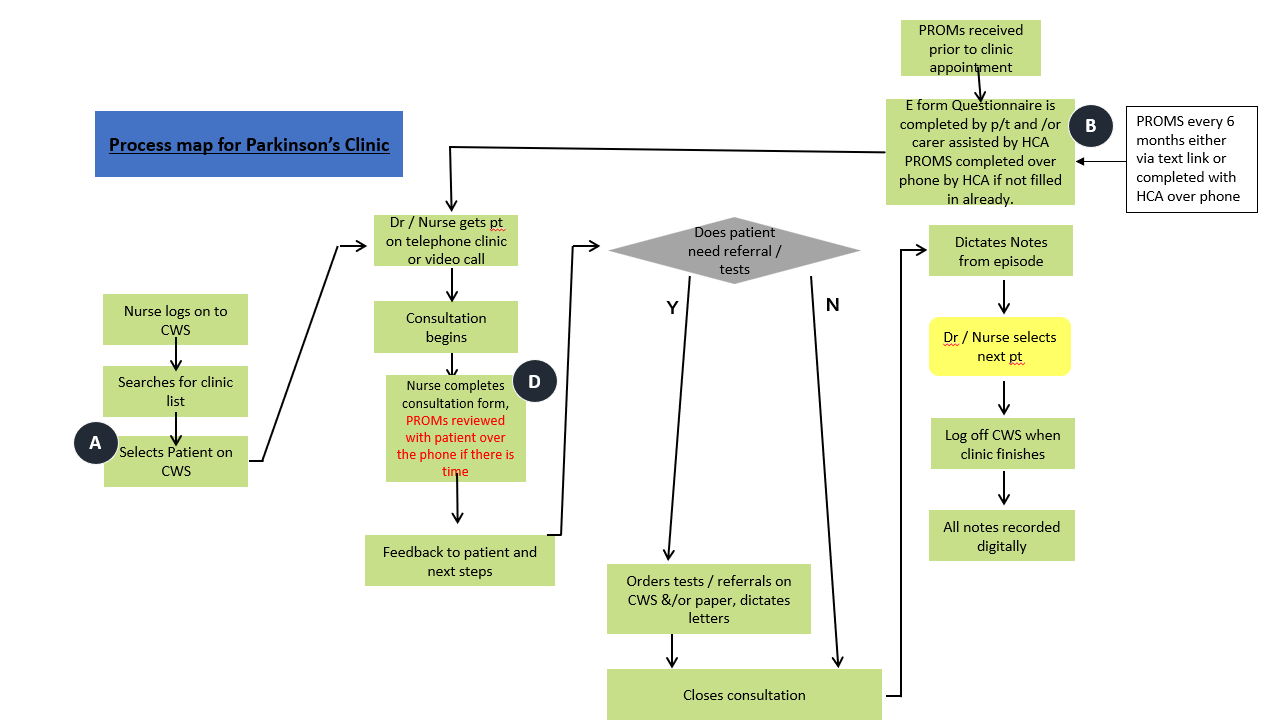


Figure S2.10 Pandemic Process Maps – Parkinson’s disease

Key: PROMs – Patient Reported Outcome Measures; HCA – Healthcare assistant; pt – patient; CWS – Clinical Work Station


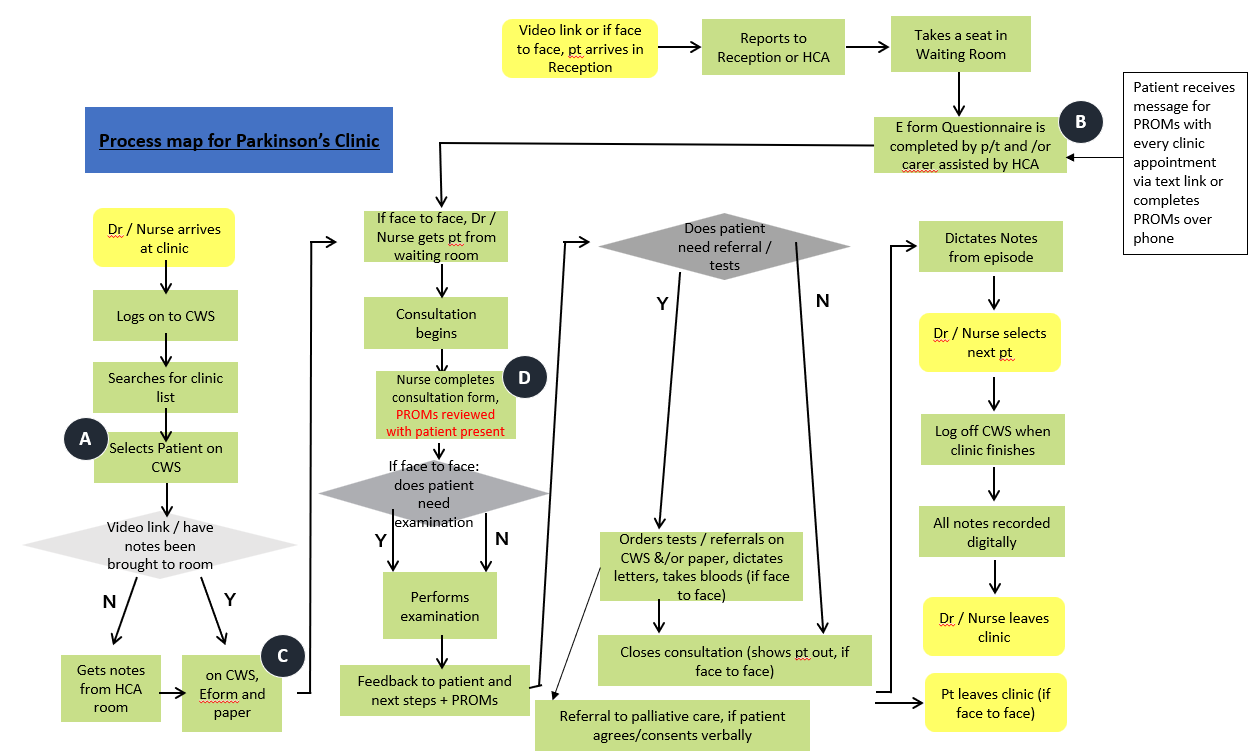


Figure S2.11. Post-pandemic Process Maps – Parkinson’s disease

Key: PROMs – Patient Reported Outcome Measures; HCA – Healthcare assistant; pt – patient; CWS – Clinical Work Station
