# Supplementary material for: A Realist Evaluation of the Implementation and Use of Patient‐Reported Outcomes in Four Value‐Based Healthcare Programmes
Source: J Adv Nurs. 2025 Jul 28;82(4):3678–701. doi: 10.1111/jan.70018 (PMC12994664; doi:10.1111/jan.70018)
Supplement: Supplementary file 3 — Data S3. [file JAN-82-3678-s002.docx]

**Supporting Information 3 – Example topic guide (patients)**

The following topic guide evolved over time as we proceeded with theory refinement and patient and staff interviews.

Table S3. Interview Schedule

| **Topics** | **Questions** | **Prompts and probes** |
| --- | --- | --- |
| **Patient context** | **Do you want to start by saying a little about you and who you are?** | Familial situation (eg: Are you married, do you have any children?), job/profession/(un)employment, etc. |
|  | **What are your experiences of living with your (named) condition?** | -When was your first diagnosis, and how long have you been taking medications? When did your symptoms start? How did you feel about it, reactions/support from family and friends, what were your concerns and worries associated with the diagnosis? Was this the first condition you were diagnosed with? Any worries or concerns about living with the health condition? |
|  | **What are your experiences in general with the NHS?** | Relations with health professionals, how do you find the support from the health service, do they understand your problems and health condition? Do you feel you had enough appointments at the beginning? What about follow-up? Any concerns you were not able to address or raise? |
| Understanding of PROMs | Can you tell me what you know about PROMs? | [participants are provided with a blank copy of PROMs questionnaire prior to the interview]. What do you think PROMs are about? What do you think is the purpose of the questionnaire? |
|  | Where did you first hear about PROMs? | What was the context, conversation with nurse/consultant/digital alerts, before/after surgery, etc? Who introduced you to PROMs and explained what PROMs were for? |
|  | Do you think the purpose of PROMs has been adequately described to you? | Did you fully understand what you were supposed to do and why? Have you had the chance to ask any questions or get further information? Do you know whom you can ask? |
|  | What do you want to get out of completing PROMs and sharing the information with your healthcare professionals? | Patient expectations, e.g., changes in treatment, feedback to professionals, better awareness of health problems and changes in health conditions etc., having a say |
|  | What do you think that PROMs are used for by your healthcare professionals? | Monitoring of health condition, better reaction to health problems and changes in health, improved organisation of treatment, e.g., reducing waiting times, avoiding unnecessary interventions and appointments, etc. |
|  | You decided not to complete the PROMs questionnaire. Can you tell me more about why not? | Did you experience difficulties completing the form? Do you think PROMs don’t make any difference to healthcare, and if so, why not? Did you have problems accessing digital forms/mail forms, questions or purpose of PROMs unclear; previous (bad?) experiences with staff/response to feedback, burdensome paperwork, lack of support for filling out PROMs, etc. |
|  | In your opinion, what is not so good about completing PROMs? | Questions or purpose of PROMs unclear; previous (bad) experiences with staff/response to feedback, difficulties with filling out the form, unclear what happens with PROMs afterwards etc., lack of free text etc. |
| Content of PROMs questionnaire | Do you feel that the PROMs questionnaire adequately describes your symptoms or condition and how they have changed over time? | Are your symptoms and health condition covered or is anything missing? Do you feel the multiple-choice questions should have other/different options? Does the questionnaire help to give an accurate picture of your health and development? In your experience, do the PROMs leave anything out that is important to you or important for your healthcare? |
|  | The questions in the PROMs focus a lot on clinical outcomes, but do you feel that other issues should be included? | For example, questions about income or social status |
|  | One important purpose of PROMs is to capture changes in health before/after surgery/during a long-term condition. Tell me, do you think this works in your case? | Do PROMs adequately capture deterioration or improvements in your health condition (before/after surgery, long-term developments), timing or frequency of PROMs etc. |
|  | PROMs questionnaires are supposed to help improving the outcome of your treatment. Have you had the feeling completing PROMs has changed your treatment? | For example, have waiting times improved, did you get different referrals etc., improved treatment according to your needs, did consultant change approach to your treatment after PROMs etc. |
|  | Have you had the chance to discuss the results of the PROMs with  your healthcare professionals? | Feedback to PROMs questionnaire, offer from professionals to discuss results of questionnaire or answer any further questions |
| Impact of PROMs and perceived outcomes | In your experience, have the priorities in your treatment changed since completing PROMs? | Change in treatment options, offer or discussion of a different treatment pathway, stronger focus on outcome/patient needs |
|  | How do you think PROMs will benefit you/your loved ones/ABUHB? | Stronger focus on patient’s/family’s views and experiences, patients as experts in their own health condition, better targeted support for care at home etc.? |
|  | Were there any unexpected consequences for you as a result of filling out the questionnaire? If so, can you say more about it? | When you think back what your anticipation was when filling out the PROMS, has there anything happened since then you did not expect? Did your treatment change in an unexpected way because of the PROMs questionnaire? |
|  | We believe that PROMs can make you think more about your own health. Can you say more about whether this was the case for you? How has it changed your behaviour? | Do you feel more involved in your healthcare? Different lifestyle habits, e.g. diet, exercise, daily routine etc.? Do you pay more attention to your health condition/symptoms etc.? More confidence in talking about health with professionals? |
|  | Do you feel monitoring of your health has improved as an outcome of PROMs? | For example, have your appointments changed, are you seen more/less often? Do you feel professionals are well informed about the progress of your health? |
|  | Do you believe there are any other people/ organisations/ services that could be affected by patients completing PROMs? | For example more referrals to different pathways/other services, more involvement of patient organisations etc. |
|  | For service XX, you have filled in a PROMs questionnaire previously, but then did not continue doing it. Can you explain the reasons why not? Did you experience any difficulties with the process? Have you had any negative experiences with the outcome of a PROMs evaluation? | For example, did the outcome of PROMs not meet your expectations at the beginning? Have you experienced problems with the feedback you gave? Did you think your PROMs were adequately used to evaluate your treatment? Too much bureaucracy in the process? |

Key: PROMs – Patient Reported Outcome Measures
