# Supplementary material for: A Realist Evaluation of the Implementation and Use of Patient‐Reported Outcomes in Four Value‐Based Healthcare Programmes
Source: J Adv Nurs. 2025 Jul 28;82(4):3678–701. doi: 10.1111/jan.70018 (PMC12994664; doi:10.1111/jan.70018)
Supplement: Supplementary file 4 — Data S4. [file JAN-82-3678-s009.docx]

**Supporting Information 4 – Reasons for patients not participating in the interview**

**Table S4. Reasons for patients not participating in the interview for each condition**

|  | Heart Failure | Cataract Surgery | Epilepsy | Parkinson’s Disease | Not identified |
| --- | --- | --- | --- | --- | --- |
| Lack of time | 2 | 0 |  | 0 | 1 |
| Not able to contact | 3 | 2 | 4 | 1 | 4 |
| Illness | 1 | 1 | 2 | 0 | 0 |
| Memory issues | 3 | 1 | 1 | 0 | 0 |
| Did not complete PROMs | 0 | 0 | 1 | 0 | 0 |

Key – PROMs: Patient-Reported Outcome Measures
