# Supplementary material for: A Realist Evaluation of the Implementation and Use of Patient‐Reported Outcomes in Four Value‐Based Healthcare Programmes
Source: J Adv Nurs. 2025 Jul 28;82(4):3678–701. doi: 10.1111/jan.70018 (PMC12994664; doi:10.1111/jan.70018)
Supplement: Supplementary file 5 — Data S5. [file JAN-82-3678-s003.docx]

**Supplementary File 5 – Logic Models**


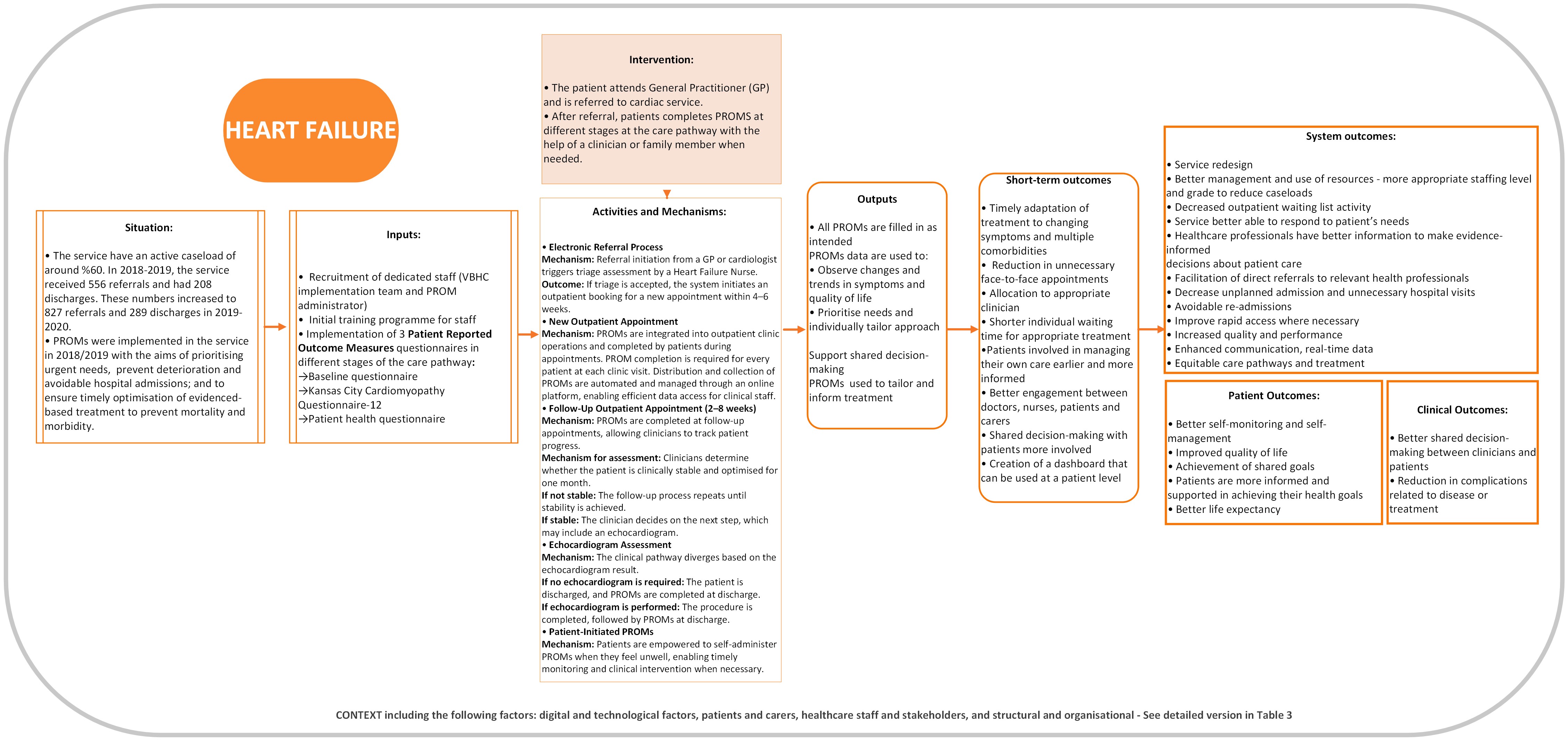


Figure S5.1 Logic Model for Heart Failure. Adapted from (Ebenso et al., 2019)

Key: PROMs – Patient Reported Outcome Measures


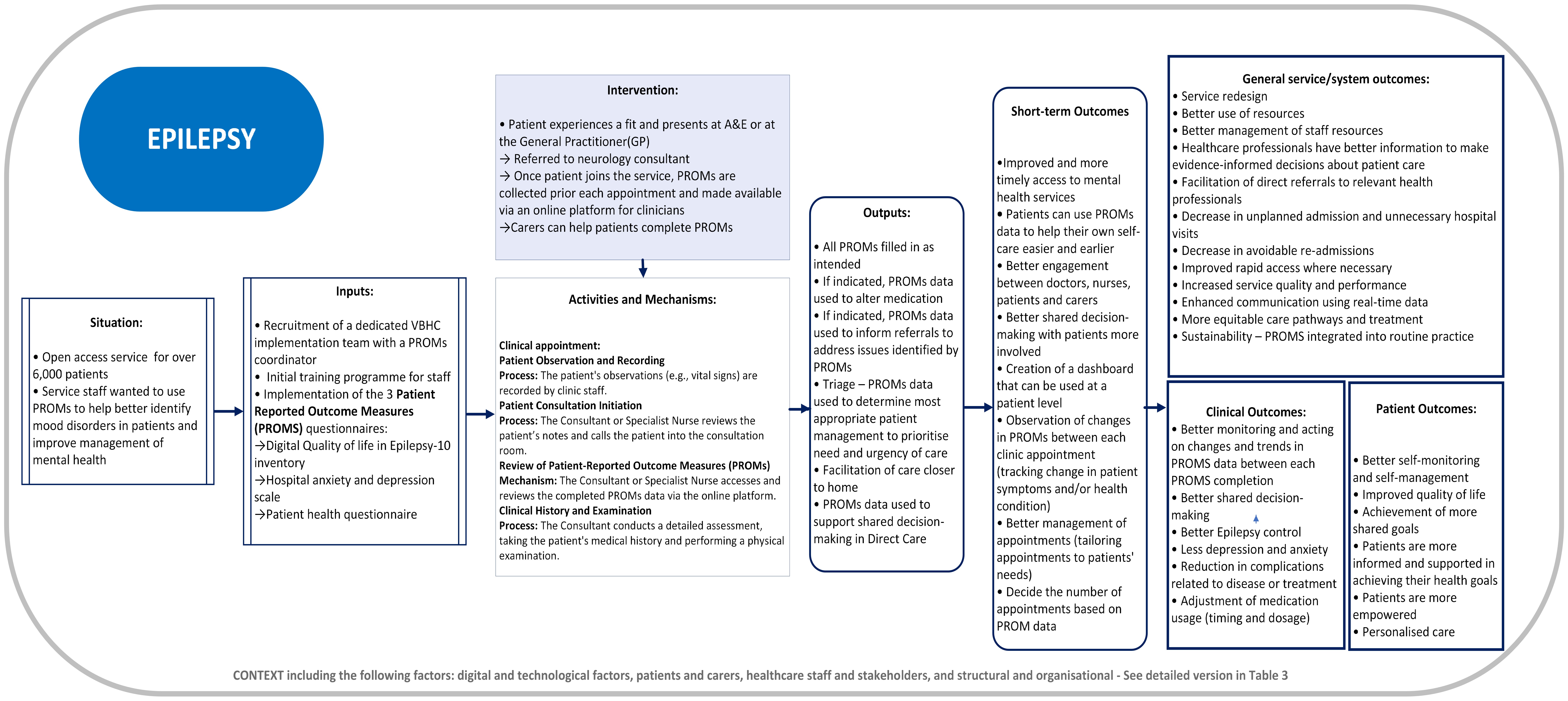


Figure S5.2 Logic Model for Epilepsy. Adapted from (Ebenso et al., 2019)

Key: PROMs – Patient Reported Outcome Measures


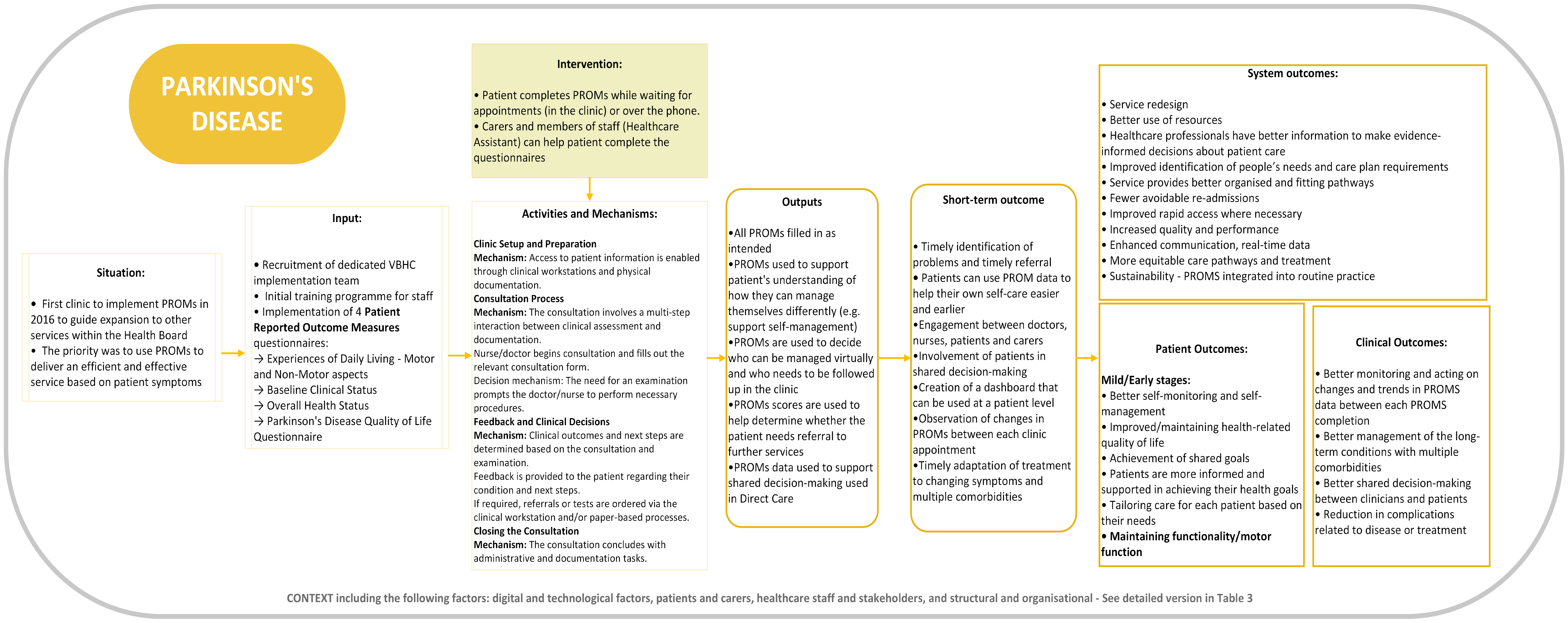


Figure S5.3 Logic Model for Parkinson’s Disease. Adapted from (Ebenso et al., 2019)

Key: PROMs – Patient Reported Outcome Measures


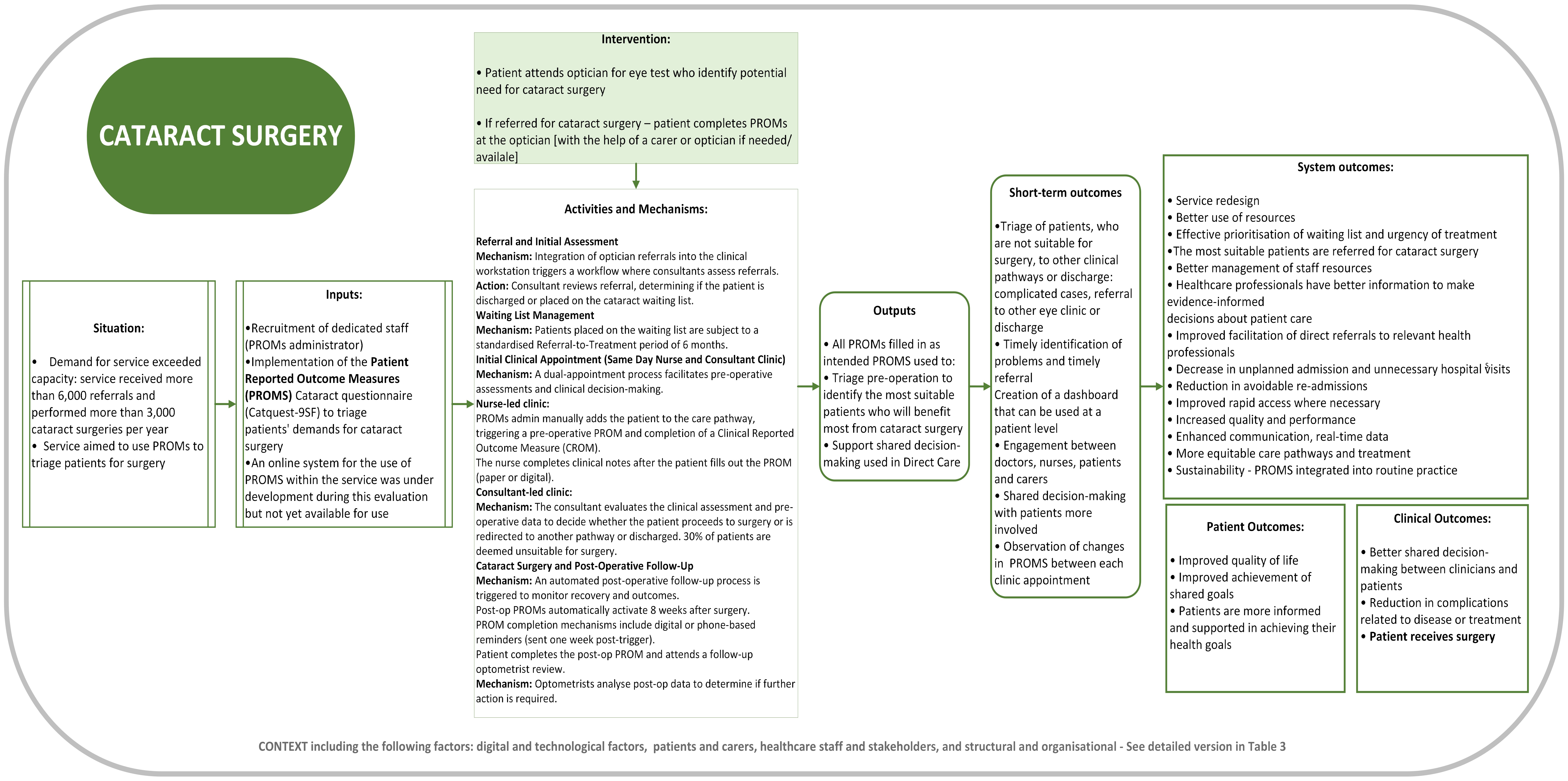


Figure S5.4 Logic Model for Cataract surgery. Adapted from (Ebenso et al., 2019)

Key: PROMs – Patient Reported Outcome Measures
