# Supplementary material for: A Realist Evaluation of the Implementation and Use of Patient‐Reported Outcomes in Four Value‐Based Healthcare Programmes
Source: J Adv Nurs. 2025 Jul 28;82(4):3678–701. doi: 10.1111/jan.70018 (PMC12994664; doi:10.1111/jan.70018)
Supplement: Supplementary file 6 — Data S6. [file JAN-82-3678-s007.docx]

**Supporting Information 6 – Participant demographics (questionnaires)**

**Table S6.** Demographics ofpatients and staff who completed online questionnaires

| **Patient Demographics** (n) | **Heart Failure**  (n = 140) | **Epilepsy**  (n = 66) | **Parkinson’s Disease**  (n = 24) |
| --- | --- | --- | --- |
| **Sex** n (%) |  |  |  |
| *Female* | 50 (36%) | 42 (80%) | 7 (29%) |
| *Male* | 89 (64%) | 11 (17%) | 17 (71%) |
| *Other* | 1 (2%) | 2 (3%) | 0 (0%) |
| **Age** n (%) |  |  |  |
| *18-35* | 2 (1%) | 30 (45%) | 0 (0%) |
| *36-50* | 8 (6%) | 21 (32%) | 2 (17%) |
| *51-70* | 55 (40%) | 12 (18%) | 9 (75%) |
| *>71* | 74 (53%) | 2 (3%) | 1 (8%) |
| **Ethnicity** n (%) |  |  |  |
| *White* | 132 (95%) | 62 (94%) | 24 (100%) |
| Black, Black British, Caribbean, or African | 2 (1%) | 0 (0%) | 0 (0%) |
| Asian or Asian British | 2 (1%) | 0 (0%) | 0 (0%) |
| *Mixed or multiple ethnic group* | 1 (<1%) | 2 (3%) | 0 (0%) |
| *Other* | 2 (1%) | 1 (2%) | 0 (0%) |
| *Prefer not to say* | 0 (0%) | 1 (2%) | 0 (0%) |
| **Years since diagnosis** n (%) |  |  |  |
| Less than a year | 12 (9%) | 0 (0%) | 0 (0%) |
| 1-2 years | 33 (24%) | 3 (5%) | 1 (4%) |
| 3-5 years | 34 (25%) | 13 (20%) | 9 (38%) |
| 6-10 years | 18 (13%) | 12 (18%) | 5 (21%) |
| >10 years | 33 (24%) | 34 (52%) | 7 (29%) |
| Prefer not to say | 3 (2%) |  | 1 (4%) |
| Not sure | 6 (4%) | 4 (6%) | 1 (4%) |
| **Participants who remember PROMs** | (n = 52) |  |  |
| Yes | 32 (23%) | 31 (47%) | 10 (42%) |
| No | 70 (50%) | 21 (32%) | 5 (21%) |
| Not sure | 20 (14%) | 11 (17%) | 7 (29%) |
| Missing | 21 (17%) | 3 (5%) | 2 (8%) |
| **Number of PROMs completed (n)** |  |  |  |
| One | 7 (13%) | 6 (14%) | 2 (12%) |
| Two | 11 (21%) | 3 (7%) | 6 (35%) |
| Three | 6 (12%) | 4 (10%) | 1 (6%) |
| Four | 3 (6%) | 3 (7%) | 2 (12%) |
| Five + | 2 (4%) | 3 (7%) | 0 (0%) |
| Do not recall completing PROM | 7 (13%) | 9 (21%) | 1 (6%) |
| Not sure | 16 (30%) | 14 (33%) | 5 (29%) |
| **Participant who used PROMs during appointments** |  |  |  |
| Very large amount | 1 (2%) | 0 (0%) | 0 (0%) |
| Large amount | 3 (6%) | 0 (0%) | 2 (12%) |
| Moderate amount | 11 (21%) | 3 (7%) | 3 (18%) |
| Small amount | 5 (9%) | 6 (14%) | 1 (6%) |
| Not at all | 19 (36%) | 22 (52%) | 6 (35%) |
| Not sure / I don’t recall completing a PROM | 13 (25%) | 11 (26%) | 5 (29%) |
| **Number of patients for who clinicians have used PROMs during appointments (n)** |  |  |  |
| Very large amount | 1 (2%) | 0 (0%) | 1 (6%) |
| Large amount | 3 (6%) | 1 (2%) | 0 (0%) |
| Moderate amount | 11 (21%) | 1 (2%) | 2 (12%) |
| Small amount | 5 (9%) | 5 (12%) | 2 (12%) |
| Not at all | 19 (36%) | 23 (55%) | 7 (41%) |
| Not sure / I don’t recall completing a PROM | 13 (25%) | 12 (29%) | 5 (29%) |
| **How much do PROMs improve care (n)** | **(n = 53)** |  |  |
| Very large amount | 5 (9%) | 1 (2%) | 1 (6%) |
| Large amount | 8 (15%) | 1 (2%) | 3 (18%) |
| Moderate amount | 11 (21%) | 1 (2%) | 3 (18%) |
| Small amount | 9 (17%) | 8 (19%) | 1 (6%) |
| Not at all | 7 (13%) | 20 (48%) | 3 (18%) |
| Not sure / I don’t recall completing a PROM | 13 (13%) | 9 (21%) | 6 (35%) |
| Missing | 5 (9%) | 2 (5%) | 0 (0%) |
| **Would it make a difference if PROMs were removed (n)** |  |  |  |
| Very large amount | 3 (6%) | 1 (2%) | 1 (6%) |
| Large amount | 7 (12%) | 3 (7%) | 1 (6%) |
| Moderate amount | 6 (11%) | 0 (0%) | 4 (24%) |
| Small amount | 8 (15%) | 7 (17%) | 0 (0%) |
| Not at all | 9 (17%) | 19 (45%) | 5 (29%) |
| Not sure / I don’t recall completing a PROM | 14 (26%) | 10 (24%) | 6 (35%) |
| Missing | 5 (9%) | 2 (5%) | 0 (0%) |

Key: PROMs – Patient-reported Outcome Measures
