# Supplementary material for: A Realist Evaluation of the Implementation and Use of Patient‐Reported Outcomes in Four Value‐Based Healthcare Programmes
Source: J Adv Nurs. 2025 Jul 28;82(4):3678–701. doi: 10.1111/jan.70018 (PMC12994664; doi:10.1111/jan.70018)
Supplement: Supplementary file 7 — Data S7. [file JAN-82-3678-s001.docx]

**Supplementary File 7 – Annotated logic models**

**
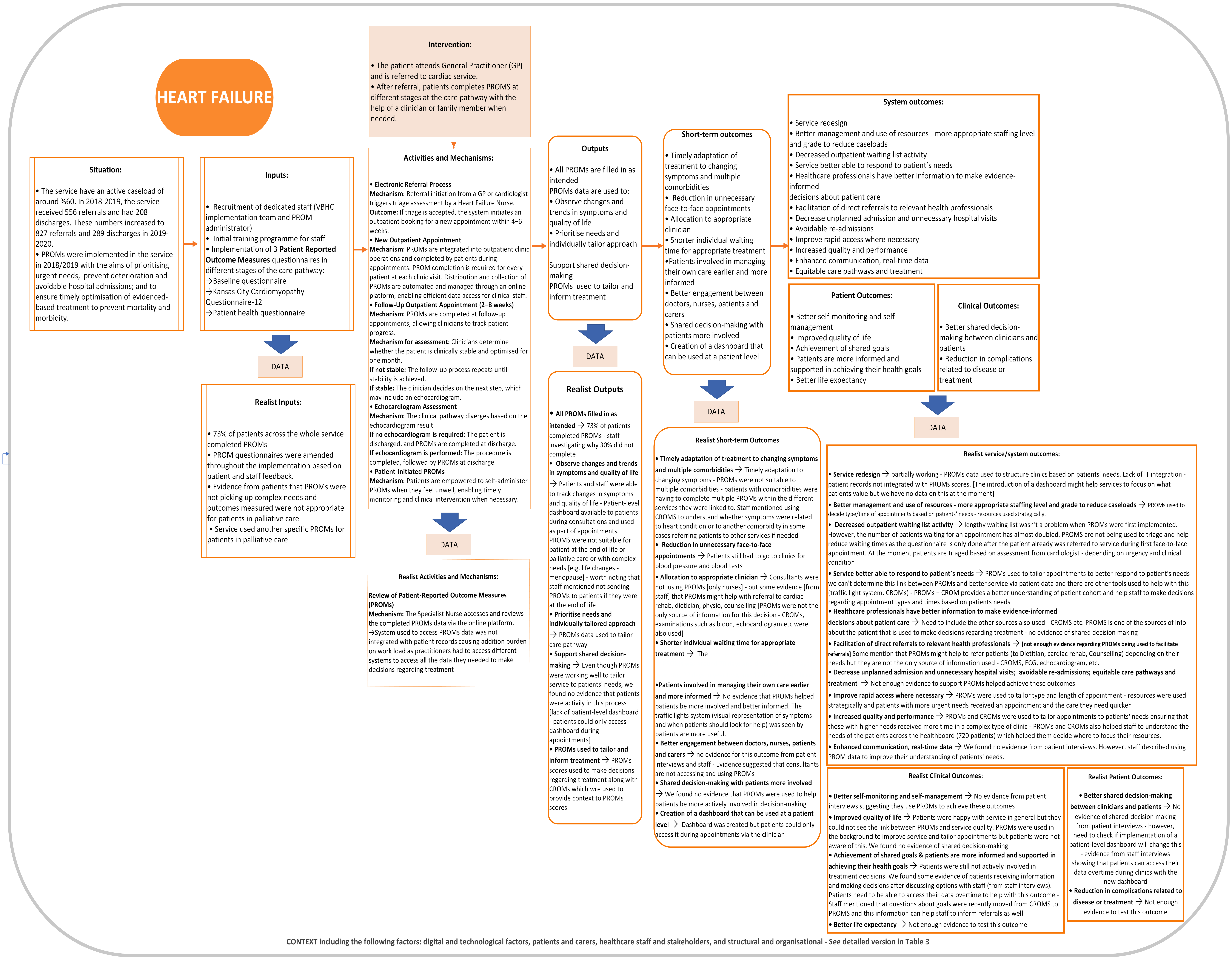
**

Figure S7.1 Annotated logic model – Heart Failure. Adapted from (Ebenso et al., 2019)

Key: PROMs – Patient Reported Outcome Measures

**
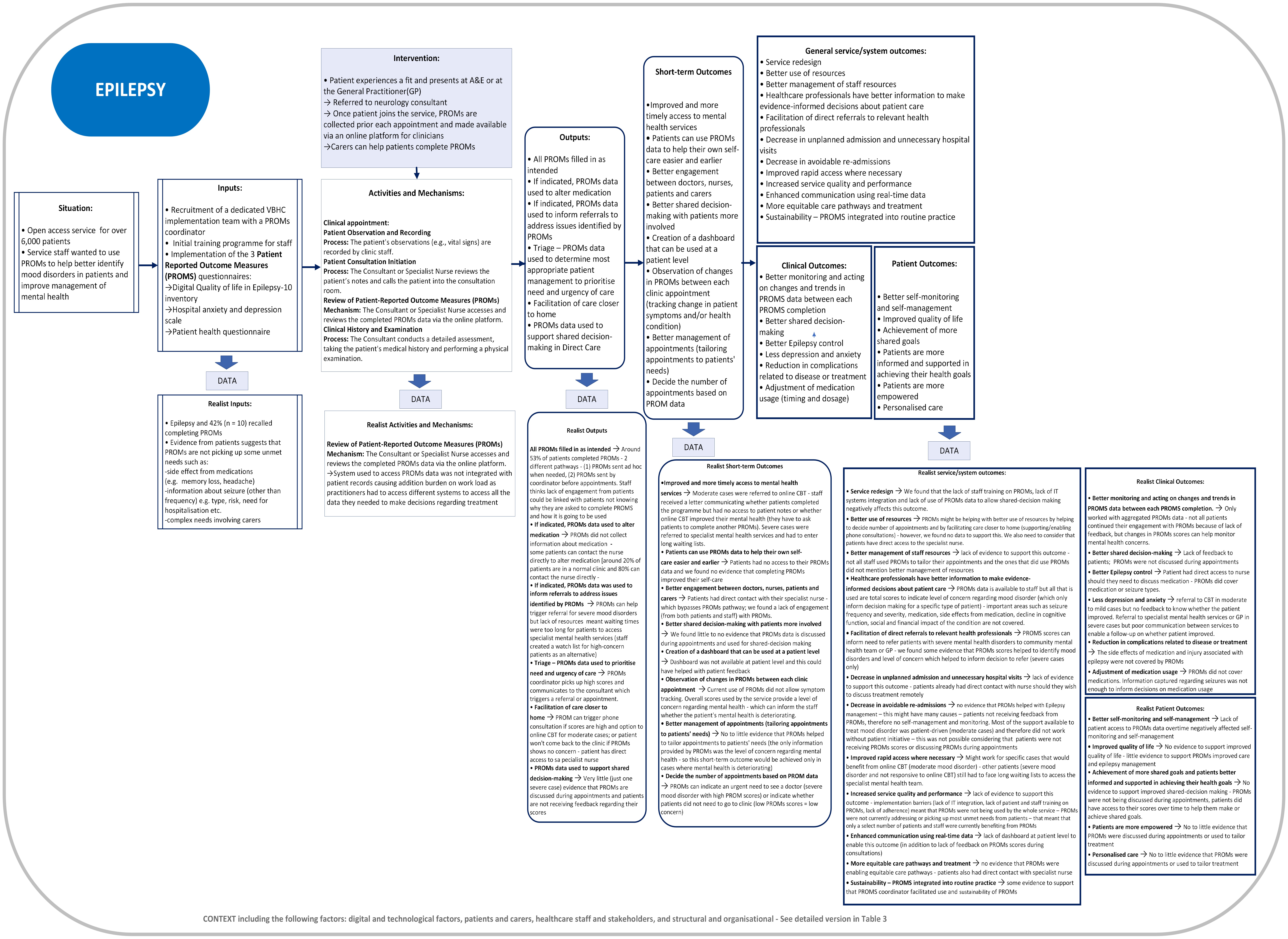
**

Figure S7.2 Annotated logic model – Epilepsy. Adapted from (Ebenso et al., 2019)

Key: PROMs – Patient Reported Outcome Measures


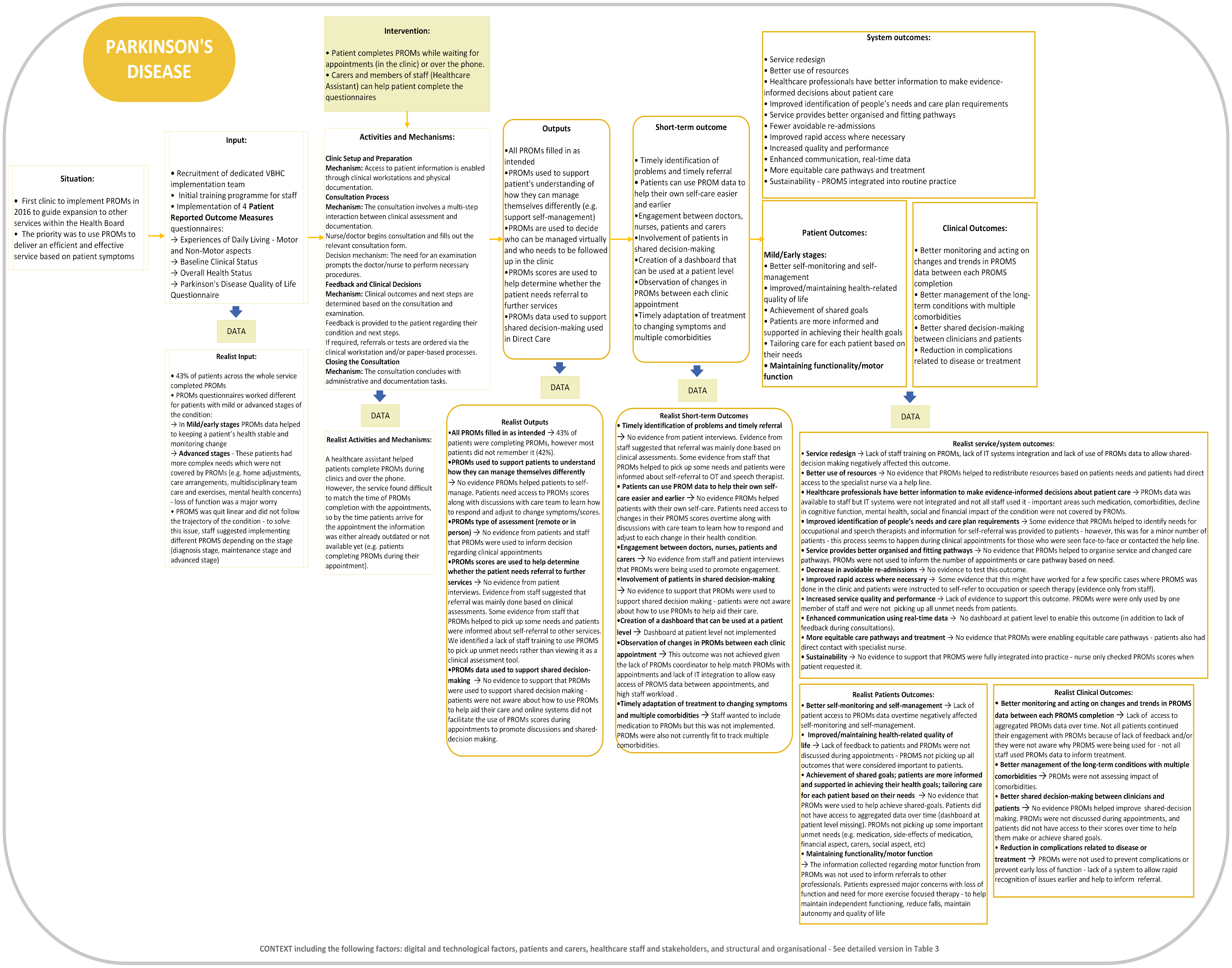
Figure S7.3 Annotated logic model – Parkinson’s Disease. Adapted from (Ebenso et al., 2019)

Key: PROMs – Patient Reported Outcome Measures


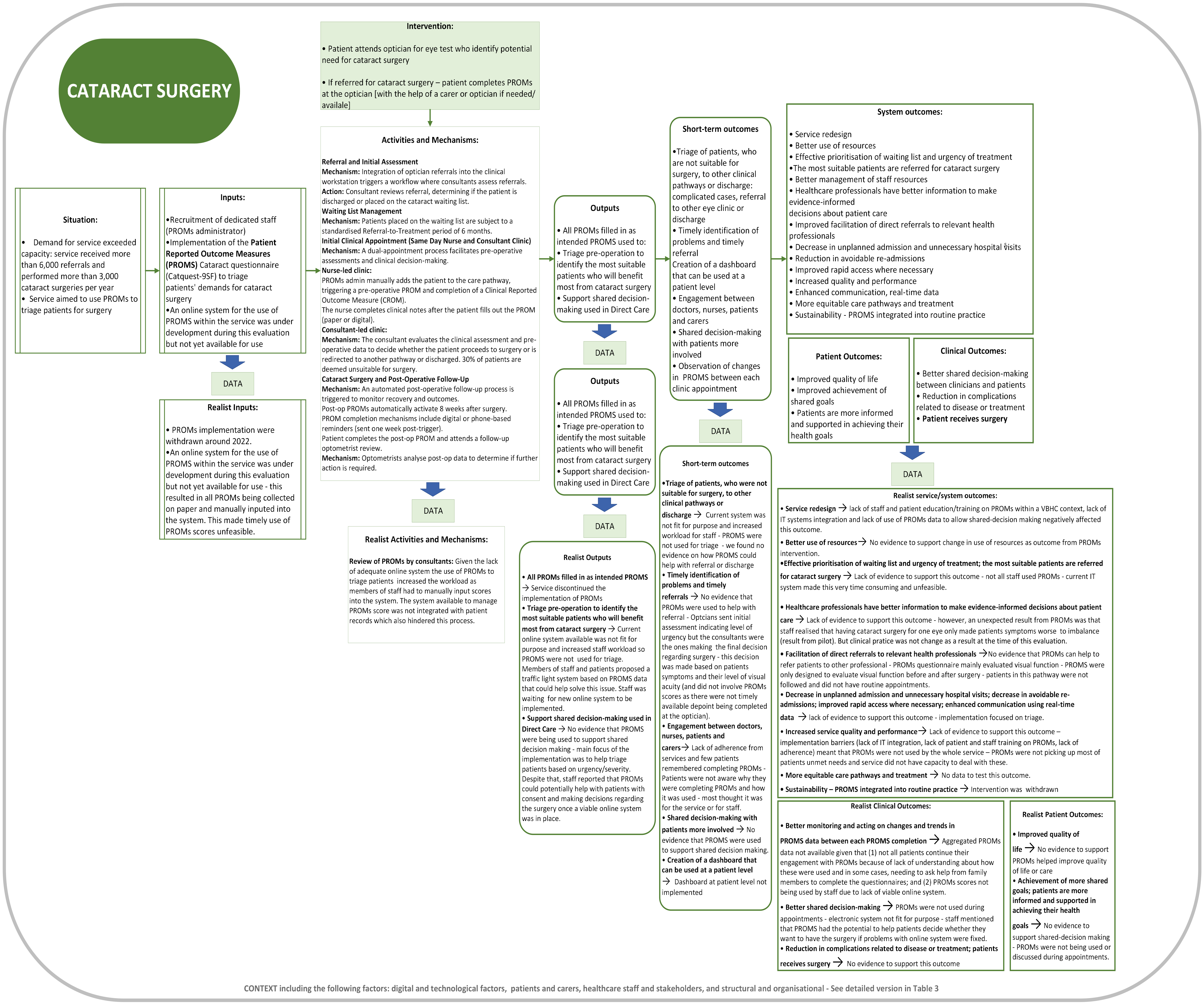


Figure S7.4 Annotated logic model – Cataract surgery. Adapted from (Ebenso et al., 2019)

Key: PROMs – Patient Reported Outcome Measures
