# Supplementary material for: A Realist Evaluation of the Implementation and Use of Patient‐Reported Outcomes in Four Value‐Based Healthcare Programmes
Source: J Adv Nurs. 2025 Jul 28;82(4):3678–701. doi: 10.1111/jan.70018 (PMC12994664; doi:10.1111/jan.70018)
Supplement: Supplementary file 8 — Data S8. [file JAN-82-3678-s006.docx]

**Supplementary File 8 – IF, THEN, LEADING TO**

**
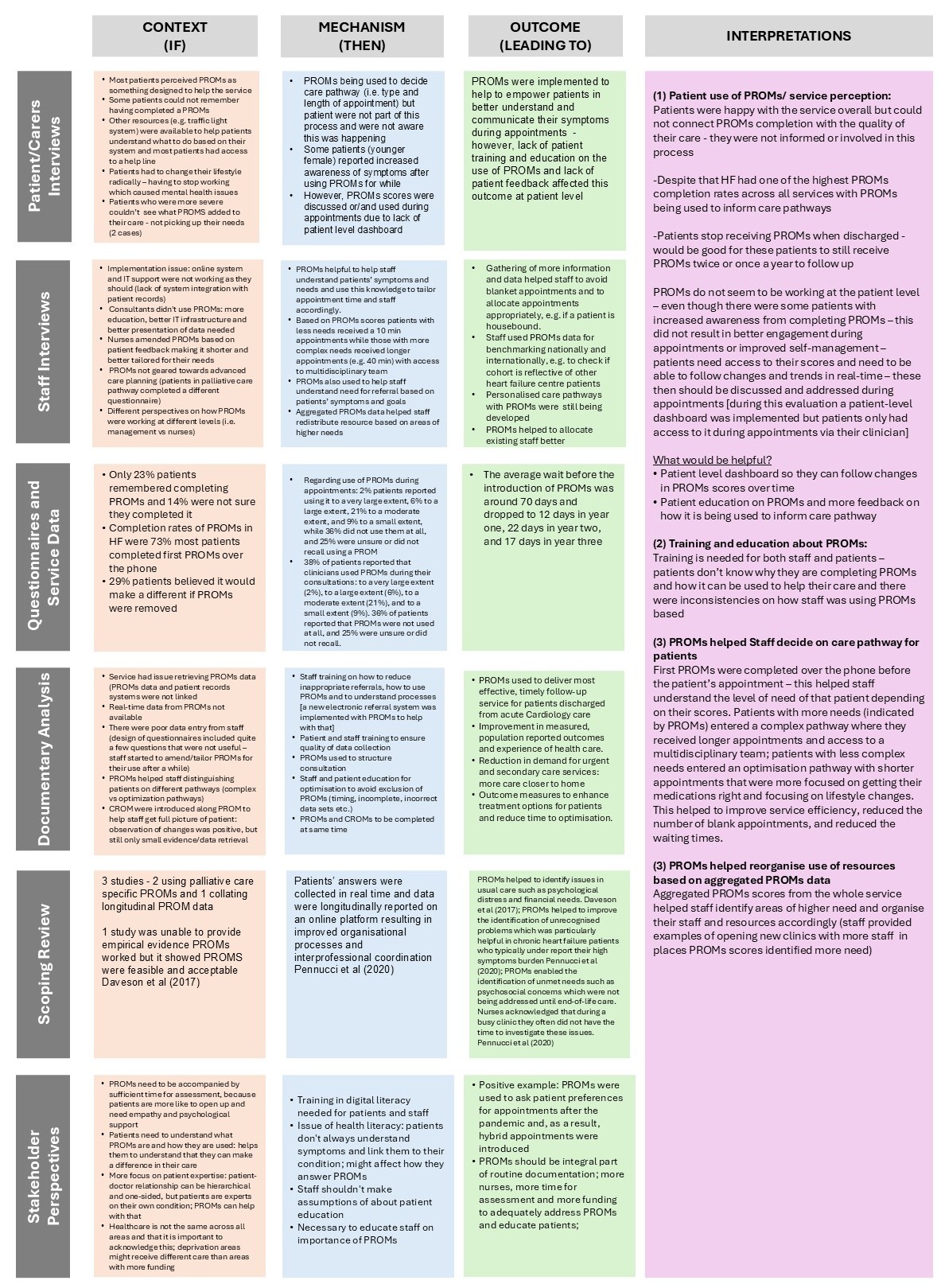
**

Figure S8. Example of IF, THEN, LEADING TO framework used to integrate the different sources of data used in the synthesis for the Heart Failure service.

Key: PROMs – Patient Reported Outcome Measures
