# Supplementary material for: A Realist Evaluation of the Implementation and Use of Patient‐Reported Outcomes in Four Value‐Based Healthcare Programmes
Source: J Adv Nurs. 2025 Jul 28;82(4):3678–701. doi: 10.1111/jan.70018 (PMC12994664; doi:10.1111/jan.70018)
Supplement: Supplementary file 9 — Data S9. [file JAN-82-3678-s004.docx]

**Supplementary File 9 – Shared Decision-making Model**

Figure S9. NHS England shared decision-making (SDM) Implementation Framework (Hendry, Turner, Lorgelly, & Woodburn, 2012)

**References**

Hendry, G. J., Turner, D. E., Lorgelly, P. K., & Woodburn, J. (2012). Room for improvement: patient, parent, and practitioners&#x2019; perceptions of foot problems and foot care in juvenile idiopathic arthritis. *93*, 2062.
